# CTCF and EGR1 suppress breast cancer cell migration through transcriptional control of Nm23-H1

**DOI:** 10.1038/s41598-020-79869-9

**Published:** 2021-01-12

**Authors:** Ka Ming Wong, Jiaxing Song, Yung H. Wong

**Affiliations:** 1 Division of Life Science and the Biotechnology Research Institute, Hong Kong University of Science and Technology, Clear Water Bay, Kowloon, Hong Kong; 2 State Key Laboratory of Molecular Neuroscience, Hong Kong University of Science and Technology, Clear Water Bay, Kowloon, Hong Kong

**Keywords:** Breast cancer, Gene regulation, Transcription

## Abstract

Tumor metastasis remains an obstacle in cancer treatment and is responsible for most cancer-related deaths. Nm23-H1 is one of the first metastasis suppressor proteins discovered with the ability to inhibit metastasis of many cancers including breast, colon, and liver cancer. Although loss of Nm23-H1 is observed in aggressive cancers and correlated with metastatic potential, little is known regarding the mechanisms that regulate its cellular level. Here, we examined the mechanisms that control Nm23-H1 expression in breast cancer cells. Initial studies in aggressive MDA-MB-231 cells (expressing low Nm23-H1) and less invasive MCF-7 cells (expressing high Nm23-H1) revealed that mRNA levels correlated with protein expression, suggesting that transcriptional mechanisms may control Nm23-H1 expression. Truncational analysis of the Nm23-H1 promoter revealed a proximal and minimal promoter that harbor putative binding sites for transcription factors including CTCF and EGR1. CTCF and EGR1 induced Nm23-H1 expression and reduced cell migration of MDA-MB-231 cells. Moreover, CTCF and EGR1 were recruited to the Nm23-H1 promoter in MCF-7 cells and their expression correlated with Nm23-H1 levels. This study indicates that loss of Nm23-H1 in aggressive breast cancer is apparently caused by downregulation of CTCF and EGR1, which potentially drive Nm23-H1 expression to promote a less invasive phenotype.

## Introduction

The major cause of cancer-related death is attributed to metastasis, a process in which cancer cells spread to distant parts of the body to form new tumors. The metastatic cascade involves multiple steps and starts from the detachment of cancer cells from the primary tumor site, intravasation into the vascular or lymphatic system, to the extravasation at the secondary site where cancer cells continue to proliferate. The activation or inactivation of numerous genes during this process provided hints into the molecular basis of the disease. In particular, a group of genes collectively known as metastasis suppressors have the ability to inhibit metastasis and are often downregulated in aggressive cancers^1^.

The murine *Nme1* gene encodes the first metastasis suppressor protein with reduced expression in highly metastatic murine melanoma^2^. The human equivalent protein, Nm23-H1, has the ability to inhibit the metastatic potential of human cancers without blocking primary tumor growth^3,4^. Human cohort studies have also revealed a strong correlation between reduced Nm23-H1 protein levels and high metastatic potential in breast, colorectal, gastric, liver, melanoma, and prostate cancers^5^. Nm23-H1 suppresses multiple steps of the metastatic cascade including intravasation, extravasation, as well as colonization of cancer cells at the secondary site^1^. Diverse studies revealed the intrinsic activities of Nm23-H1 that potentially mediate its metastasis suppressor function, and include nucleoside diphosphate kinase activity^6^, histidine protein kinase activity^7^, and 3′–5′ exonuclease activity^8^. In addition, Nm23-H1 interacts with a plethora of proteins that further define its metastasis-related functions^9^. Despite high sequence similarity, the closely related Nm23-H2 isoform associates with distinct interaction partners to assume different roles in metastasis suppression of several cancers^10^. Accumulating evidence also suggest that Nm23-H2 can regulate numerous signaling pathways linked to tumorigenesis in solid tumors and hematological malignancies^11,12^.

In breast cancer, the expression of Nm23-H1 is negatively correlated with metastatic potential and poor clinical outcome^13–15^. Nm23-H1 expression was reduced in a panel of breast cancer carcinomas without harboring coding sequence mutations and was correlated with poor survival^16^. Unlike genes that are downregulated in cancer because of mutations in the coding sequence, mutations in the *NME1* gene are rare and until now only the S120G mutation in neuroblastoma patients has been reported^17^. Insertions and deletions in the *NME1* gene are also absent according to the COSMIC database^18^. Further clinical data indicate a negative correlation between metastatic potential and Nm23-H1 expression at both the transcript and protein levels in breast cancer^19,20^, suggesting that transcriptional mechanisms is a major contributor to the downregulation of Nm23-H1 in aggressive breast cancer. Alternative mechanisms regulating Nm23-H1 protein expression include cathepsin-induced degradation pathways^21^ and the action of miRNAs^22,23^, but these would not contribute to the reduction of transcript levels as observed in metastatic breast cancers.

Although several reports have provided subtle hints into the transcriptional control of Nm23-H1^24,25^, such models remain poorly defined and cannot explain the downregulation of Nm23-H1 in aggressive cancers. The discovery of transcriptional mechanisms may provide additional options for therapeutic intervention that aims to control Nm23-H1 expression and the metastatic disease. In fact, many small molecules have the ability to elevate the expression of a single, or multiple metastasis suppressors, which presumably involves complex transcriptional and post-transcriptional mechanisms^26^.

In this study, we analyzed the promoter region of *NME1* using bioinformatic tools and reporter genes to identify novel transcription factors regulating Nm23-H1 expression in breast cancer cells. Several binding sites were found for CTCF and EGR1, which correlated with Nm23-H1 protein levels in less invasive MCF-7 cells and both transcription factors were able to drive Nm23-H1 expression in highly aggressive MDA-MB-231 cells. The transcriptional control of Nm23-H1 by CTCF and EGR1 provides a mechanism for their ability to inhibit the metastatic process of breast cancer cells.

## Results

### Transcriptional activity of *NME1* promoter is reduced in aggressive breast cancer

**Figure 1:**
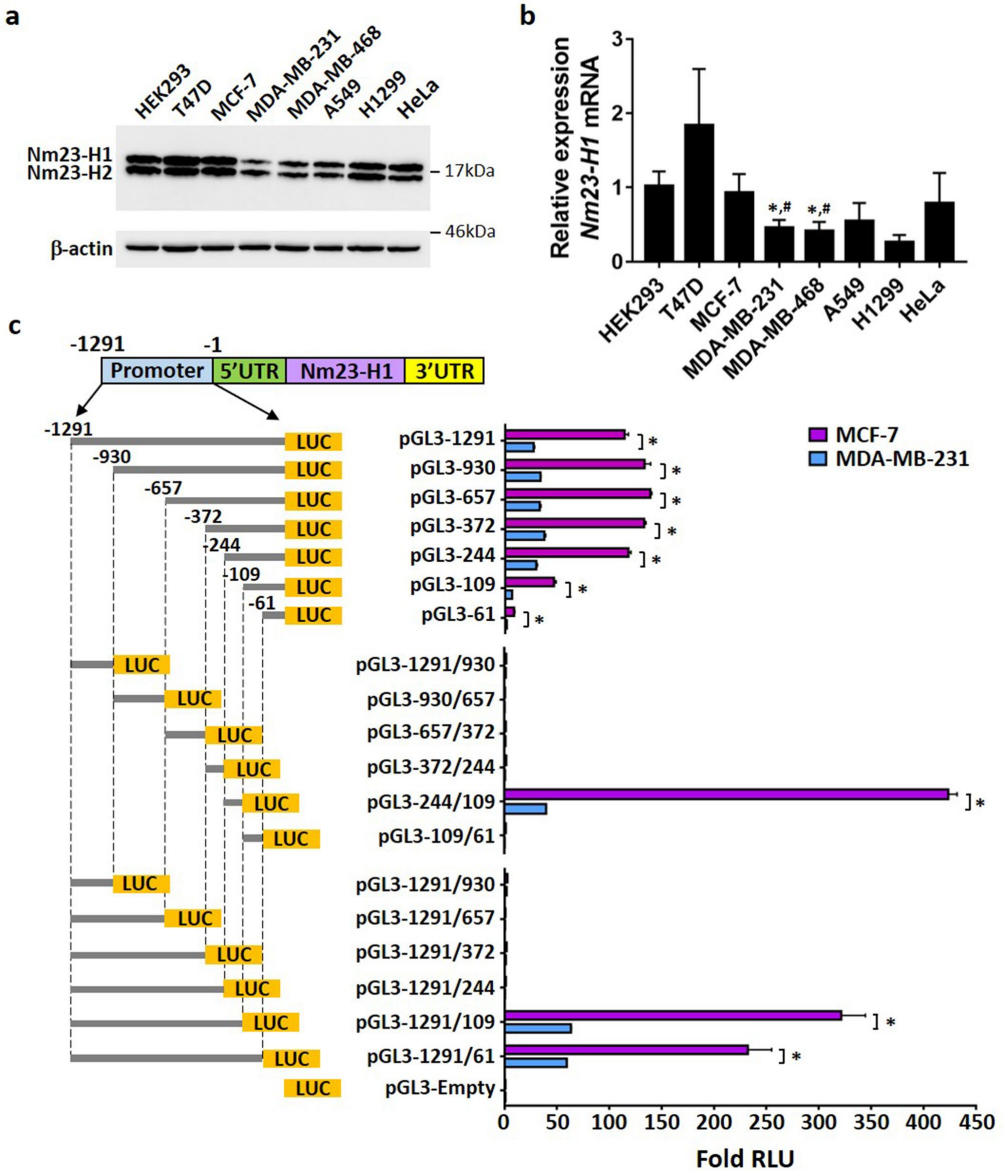
Analysis of Nm23-H1 protein, mRNA, and promoter activity in breast cancer cell lines. Confluent cell cultures were lysed and analyzed for Nm23-H1 expression by Western blot with corresponding antibodies (**a**) or RT-qPCR (**b**). (**c**) MCF-7 and MDA-MB-231 cells were transiently transfected with Nm23-H1 promoter constructs and pRL-TK control vector and lysed 48 h after transfection. Luciferase activities were measured using the Dual-Luciferase Reporter Assay System (Promega). RLU values of reference constructs: MCF-7/pGL3-Empty, *Firefly* luciferase (10,431 ± 311), *Renilla* luciferase (92,978 ± 1766); MDA-MB-231/pGL3-Empty, *Firefly* luciferase (1967 ± 75), *Renilla* luciferase (10,371 ± 183). Luciferase activities were calculated using *firefly* luciferase values normalized to *renilla* luciferase values. Data represent the mean and standard deviation of three independent trials. *p < 0.05.

Low expression of Nm23-H1 is correlated with metastasis and poor clinical outcome in many cancer types including melanoma, breast, colon and liver cancer. Nm23-H1 is abundantly expressed in less invasive and early stage cancers, while the expression is lost in aggressive cancer subtypes^9^. In agreement with these findings, the protein expression of Nm23-H1 in the highly aggressive MDA-MB-231 and MDA-MB-468 breast cancer cells is significantly lower as compared to its expression in the less metastatic MCF-7 and T47D breast cancer cells (Fig. 1a; upper band). Transcript levels of Nm23-H1 were also observed to correspond to protein expression levels (Fig. 1b), suggesting that transcriptional mechanisms may be responsible for the loss of Nm23-H1. This pattern was not observed for metastatic H1299 lung cancer cells versus the less metastatic A549 variant of lung cancer cells, consistent with several studies indicating a positive correlation of Nm23-H1 expression with lung cancer metastasis^27^. The protein expression of the Nm23-H2 isoform was also strongly downregulated in MDA-MB-231 and MDA-MB-468 cells (Fig. 1a; lower band).

To search for transcriptional mechanisms controlling Nm23-H1 expression, luciferase constructs were generated to examine the activity of different promoter segments. A promoter region of *NME1* (from − 1291 bp to − 1 bp) upstream of the transcription start site (TSS) was amplified from genomic DNA and cloned into the pGL3-Basic vector to generate pGL3-1291. Promoter bashing was performed to generate a series of 5′ end and 3′ end truncated constructs. In MCF-7 cells transiently transfected with the various constructs, a strong induction of luciferase was observed for the pGL3-109 construct, which encompasses the region from − 109 bp to − 1 bp and potentially represents the minimal promoter region driving basal expression (Fig. 1c). The largest increase in luciferase activity was detected when comparing the pGL3-244 and pGL3-109 constructs, indicating that the most significant elements driving Nm23-H1 promoter activity lie between − 244 bp and − 109 bp upstream of the TSS and represent the proximal promoter region. Double truncation at both the 5′ and 3′ ends confirmed the significance of the proximal promoter as shown by the activity of the pGL3-244/109 construct, which lacks the minimal promoter region and displayed the highest luciferase activity among all constructs. Further 3′ end promoter truncations showed that the luciferase activity of pGL3-1291/61 was lower than that of pGL3-1291/109, indicating that repressive elements may reside in the region between − 109 bp and − 61 bp. More importantly, the activity of promoter constructs in MDA-MB-231 cells were significantly lower when compared to MCF-7 cells, implying that downregulation of Nm23-H1 in highly metastatic breast cancer is apparently caused by reduced transcriptional activity at the Nm23-H1 promoter. Promoter constructs were also analyzed in non-malignant HEK293 cells expressing detectable Nm23-H1 mRNA and protein levels (Fig. 1a,b), which generated a similar pattern of activity (Supplementary Fig. S1).

### Identification of transcription factors interacting with the *NME1* promoter

**Figure 2:**
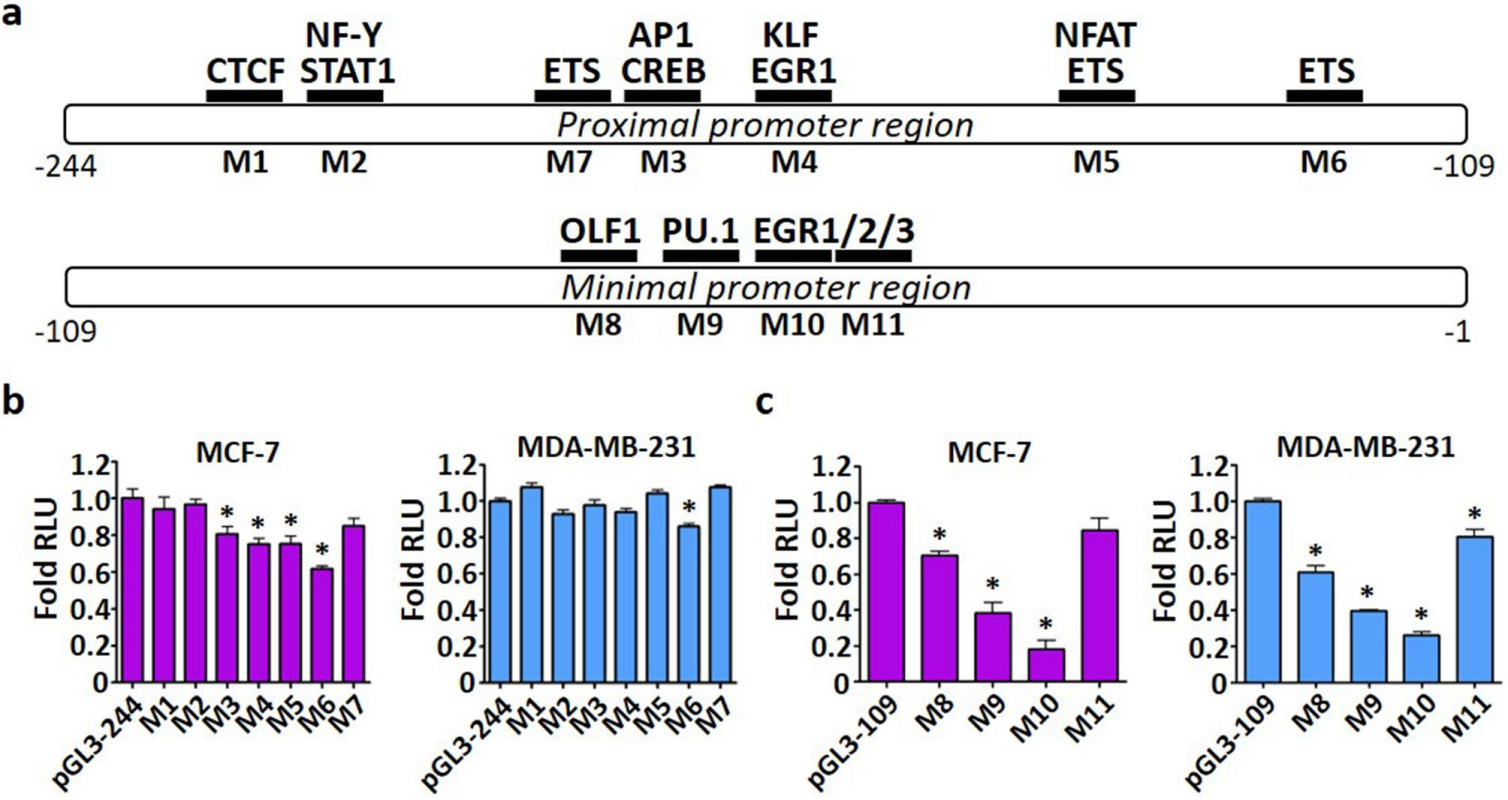
In silico screening of transcription factor binding sites. (**a**) The proximal (− 244 to − 109 bp) and minimal promoter (− 109 to − 1 bp) region of the Nm23-H1 promoter were analyzed in MatInspector (matrix similarity threshold > 0.8) and TRANSFAC (matrix score threshold > 0.8) for potential transcription factor binding events. Consistently predicted transcription factors and corresponding binding sites 1–11 were selected for mutagenesis. Mutants of the proximal promoter (**b**) and minimal promoter (**c**) were transiently transfected in MCF-7 or MDA-MB-231 cells and lysed 48 h after transfection. Luciferase assays were performed using the Dual-Luciferase Reporter Assay System (Promega). RLU values of reference constructs: MCF-7/pGL3-244, *Firefly* luciferase (2,749,183 ± 106,184), *Renilla* luciferase (208,951 ± 7441); MCF-7/pGL3-109, *Firefly* luciferase (689,809 ± 49,773), *Renilla* luciferase (131,826 ± 3867); MDA-MB-231/pGL3-244, *Firefly* luciferase (138,241 ± 401), *Renilla* luciferase (24,492 ± 1526); MDA-MB-231/pGL3-109, *Firefly* luciferase (689,809 ± 49,773), *Renilla* luciferase (131,826 ± 3867). Data represent the mean and standard deviation of three independent trials. *Significance with wild-type promoter, p < 0.05.

We next analyzed the proximal promoter region encompassing the most significant elements (from − 244 to − 109 bp) and the minimal promoter (from − 109 to − 1 bp) of *NME1* using MatInspector^28^ and TRANSFAC^29^, which are online tools that utilize algorithms to predict transcription factor binding sites. The promoter sequences of human Nm23-H1 were aligned with the macaque and mouse sequence and show 98% and 72% similarity, respectively, indicating the presence of biologically relevant DNA elements (Supplementary Fig. S2). Potential transcription factors including AP1, CTCF, CREB, EGR1, EGR2, EGR3, ETS, KLF2, KLF6, NF-Y, NFAT, OLF1, PU.1, and STAT3 were selected based on their consistent prediction by both algorithms and are shown in Fig. 2a.

To evaluate the importance of putative transcription factor binding sites, the binding sites 1–7 of the proximal promoter and 8–11 of the minimal promoter were substituted for either the *Eco*RI (GAATTC) or *Bgl*II (AGATCT) sequence, which lack any known cis-acting elements, to generate luciferase promoter mutants M1 to M11 (Table 1). Reporter studies with proximal promoter mutants M1 to M7 revealed that the *NME1* promoter activities of M3, M4, M5, and M6 were significantly reduced in MCF-7 cells (Fig. 2b). Disruption of *NME1* promoter activity by the M3 mutant is in line with previous reports that demonstrated AP-1 and CREB as transcriptional activators of *NME1*^25,30^. The proximal promoter mutants had little or no reduction in reporter activity in MDA-MB-231 cells except for mutant M6. At the minimal promoter, the reporter activities of promoter mutants M8 to M10 were decreased as compared to pGL3-109 wild-type in both MCF-7 and MDA-MB-231 cells (Fig. 2c). The M11 mutant, however, only exhibited a reduction in reporter activity in MDA-MB-231 cells. The largest suppression in reporter activity was seen with the M10 mutant. Analysis of promoter mutants in HEK293 cells revealed an additional transcription factor binding site potentially disrupted by mutant M1 (Supplementary Fig. S3). The list of transcription factors that are potentially affected by the different mutants is shown in Table 2. Interestingly, multiple binding sites were predicted for EGR1 in both the proximal and minimal promoter regions. The GA-binding protein alpha (GABPA), E74-like factor 1 (ELF1), and E74-like factor 5 (ELF5), were selected from the relatively large family of ETS transcription factors based on their binding motif similarity to the sequence of binding sites M4 and M5^31^.

**Table 1:** Mutant promoters of *NME1* and corresponding wild-type sequence, mutant sequence, and transcription factors.

Mutant	Wild-type sequence (+)	Mutant sequence (+)	Transcription factors	Core binding motif
M1	GAGC**GCCACC**TCTC	GAGC**AGATCT**TCTC	CTCF	(−)TGGC
M2	CTCT**CGGGAA**GCCA	CTCT**GAATTC**GCCA	STAT1, NF-Y	(−)TTCC, (+)GGAA
M3	AAGT**GAGTCA**GAGA	AAGT**AGATCT**GAGA	CREB, AP-1	(−)TGAC, (+)AGTC
M4	ACCC**GGGGGT**GGAG	ACCC**GAATTC**GGAG	EGR1, KLF	(+)GGGG. (+)GGGT
M5	TAAC**CGGAAA**GGTC	TAAC**GAATTC**GGTC	ETS, NFAT	(+)CGGA, (+)GGAA
M6	AGCG**CCGGAA**GTTA	AGCG**GAATTC**GTTA	ETS	(+)CGGA
M7	ACGA**AGGAAG**TGAG	ACGA**GAATTC**TGAG	ETS	(+)GGAA
M8	CCTA**CTCCCA**AGAG	CCTA**GAATTC**AGAG	OLF1	(+)TCCC
M9	CAAG**AGGAAG**CGTG	CAAG**GAATTC**CGTG	PU.1	(+)GGAA
M10	AGGA**AGCGTG**GGCG	AGGA**GAATTC**GGCG	EGR1, EGR2, EGR3	(+)GCGT
M11	GCGT**GGGCGA**GCGG	GCGT**AGATCT**GCGG	EGR1, EGR2, EGR3	(+)GGCG

Mutants M1 to M7 are located in the proximal promoter region of *NME1* between − 244 to − 109 bp, whereas mutants M8 to M11 reside in the minimal promoter region from − 109 to − 1 bp. All wild-type sequences are mutated to either the *EcoRI* (GAATTC) or *BglII* (AGATCT) sequence. Transcription factor interactions are predicted by MatInspector and TRANSFAC. Core binding motifs of corresponding transcription factors are shown at the positive strand (+) or negative strand (−).

**Table 2:** Potential transcription factors binding to the proximal and/or minimal promoter regions of *NME1* promoter.

Transcription factor	Potential binding site	Role in breast cancer metastasis	Expression in aggressive breast cancer
CTCF	M1	Suppressor^32^	Downregulation^32^
EGR1	M4, M10, M11	Suppressor^33^	Downregulation^34^
ELF1	M6	Suppressor^35^	Downregulation^36^
EGR2	M10, M11	ND	Upregulation^37^
EGR3	M10, M11	ND	ND
ELF5	M6	Suppressor^38^	Downregulation^39^
GABPA	M6	ND	Downregulation^36^
KLF2	M4	Suppressor^40^	No correlation^40^
KLF6	M4	ND	No correlation^41^
OLF1	M8	ND	ND
PU.1	M9	ND	No correlation^42^

Transcription factors predicted to bind to significant binding sites according to mutagenesis studies (Fig. 2) are shown. ELF1, ELF5, and GABPA, represent the ETS family of transcription factors. The potential role in breast cancer metastasis and expression in aggressive breast cancer are also cited.

*ND* not determined, *NA* not available.

### Several transcription factors upregulate Nm23-H1 expression in MDA-MB-231 cells

**Figure 3:**
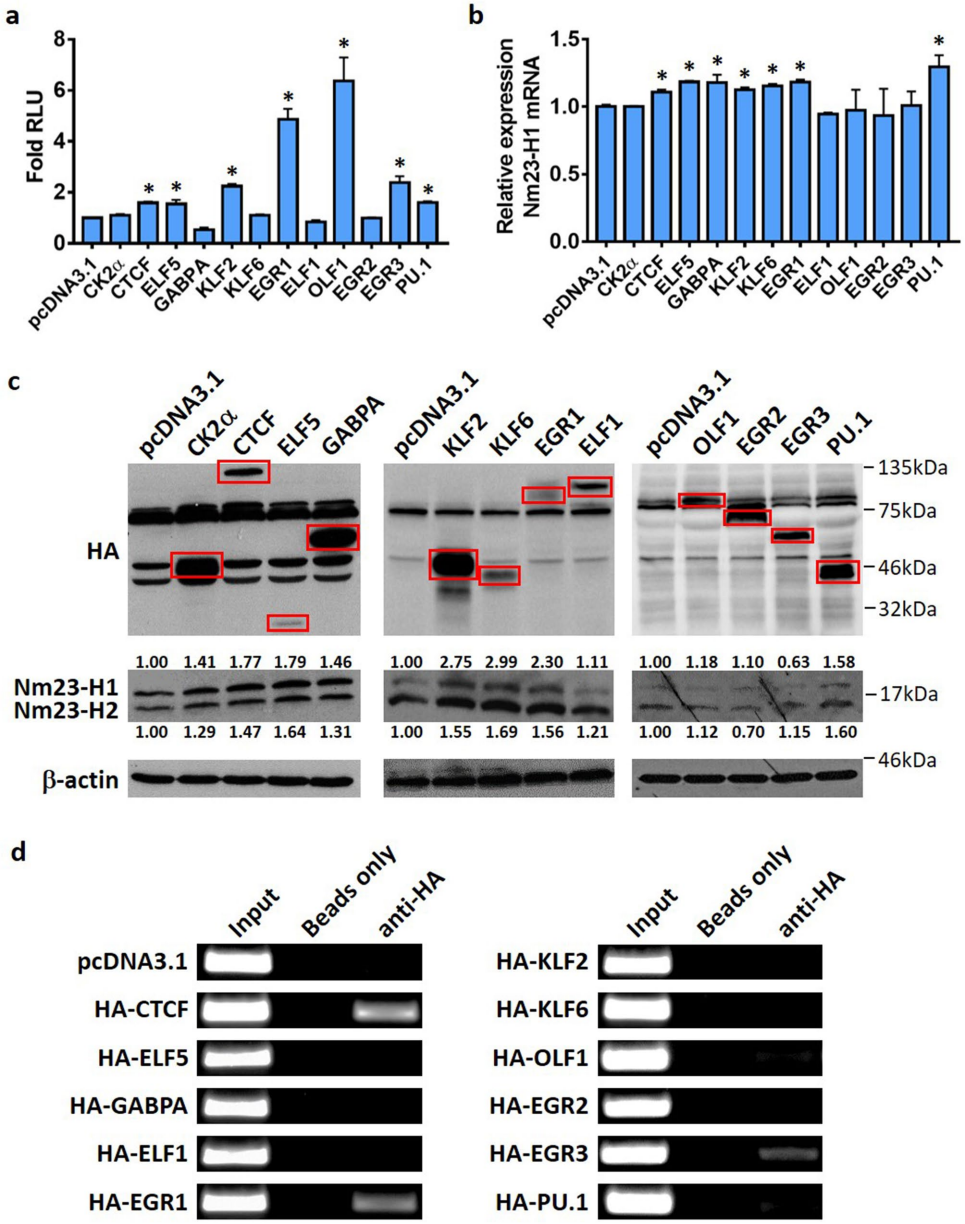
Transcription factors regulating Nm23-H1 in MDA-MB-231 cells. (**a**) MDA-MB-231 cells were transiently transfected with HA-tagged transcription factors and pGL3-1291 and lysed 48 h after transfection. Luciferase assays were performed using the Dual-Luciferase Reporter Assay System (Promega). RLU values of pGL3-1291/pcDNA3.1: *Firefly* luciferase (310,113 ± 8524), *Renilla* luciferase (60,130 ± 5091). *Significant stimulation as compared to pcDNA3.1, p < 0.05. (**b**) RNA extraction was performed with TRIzol in transiently transfected MDA-MB-231 cells, followed by cDNA synthesis and qPCR. *Significance with pcDNA3.1, p < 0.05. Data represent the mean and standard deviation of three independent trials. (**c**) Proteins were separated on 15% acrylamide gels and immunoblots were probed with corresponding antibodies. Corresponding protein bands are marked by red boxes. Quantification of Nm23-H1/H2 protein bands was performed with ImageJ. Data are shown as a representative experiment from three independent trials. (**d**) Protein-DNA interactions were crosslinked and the chromatin was sheared into 200–500 bp fragments by sonication. Complexes were pulled down by the HA-antibody and protein G agarose beads. ‘Input’ is the chromatin collected before antibody incubation. ‘Beads only’ represents the chromatin containing protein G agarose beads without antibodies and serves as a negative control. Data are shown as a representative experiment from three independent trials.

To determine if potential transcription factors can indeed regulate Nm23-H1 expression, HA-tagged transcription factors and the pGL3-1291 promoter construct were transiently transfected into MDA-MB-231 cells for luciferase reporter studies. CTCF, ELF5, EGR1, KLF2, OLF1, EGR3, and PU.1 stimulated *NME1* promoter activity, whereas repression was observed upon GABPA expression (Fig. 3a). Other transcription factors including ELF1, KLF6, and EGR2 did not alter the promoter activity. Protein and mRNA levels of Nm23-H1 were also quantified upon overexpression of transcription factors. CTCF, EGR1, ELF5, GABPA, KLF2, KLF6, and PU.1 increased Nm23-H1 transcript levels (Fig. 3b), whereas Nm23-H1 protein expression was substantially upregulated (over 50% increase) by CTCF, ELF5, KLF2, KLF6, EGR1, and PU.1 (Fig. 3c). KLF6 also induced both mRNA and protein expression, but its mechanism of upregulation may not involve transcriptional stimulation as demonstrated in reporter studies (Fig. 3a). In contrast, OLF1 and EGR3 stimulated promoter activity without enhancing mRNA or protein expression. Induction of Nm23-H1 mRNA expression by GABPA was apparently independent of promoter stimulation, while upregulation of Nm23-H1 was just below 50% at the protein level (Fig. 3c). ELF1 and EGR2 were unable to affect promoter activity and transcript levels, consistent with their limited potential to increase protein expression. The CTCF activator protein casein kinase 2 alpha (CK2α), which activates CTCF by phosphorylation^43^, slightly increased Nm23-H1 protein expression without affecting mRNA level and promoter activity. Albeit weaker than Nm23-H1, upregulation of Nm23-H2 protein levels was also clearly evident upon overexpression of ELF5, KLF2, KLF6, EGR1, and PU.1 (Fig. 3c). Potential transcription factors that stimulated *NME1* promoter activity, transcript level, as well as protein expression include CTCF, EGR1, ELF5, KLF2, and PU.1, suggesting that these factors may bind to the *NME1* promoter to activate transcription.

To confirm their recruitment to the Nm23-H1 promoter, ChIP assays were performed in MDA-MB-231 cells overexpressing the transcription factors. The immunoprecipitated complex was analyzed for the presence of the *NME1* promoter region. PCR amplification of DNA eluted from the complex generated the correct band size for CTCF and EGR1 (Fig. 3d). Interestingly, EGR3 was also recruited to the *NME1* promoter, but the biological function of this interaction remains to be determined. ELF5, KLF2, and PU.1, on the other hand, were not able to associate with the promoter, suggesting that they may indirectly induce the transcription of *NME1* or bind to sequences beyond the analyzed promoter regions. Putative binding sites for ELF5 and PU.1 are indeed present in less active regions upstream of the proximal promoter according to bioinformatic tools (data not shown). These results suggest that CTCF and EGR1 are recruited to the *NME1* promoter to drive Nm23-H1 expression in breast cancer cells.

### CTCF and EGR1 maintain high Nm23-H1 expression in MCF-7 cells

**Figure 4:**
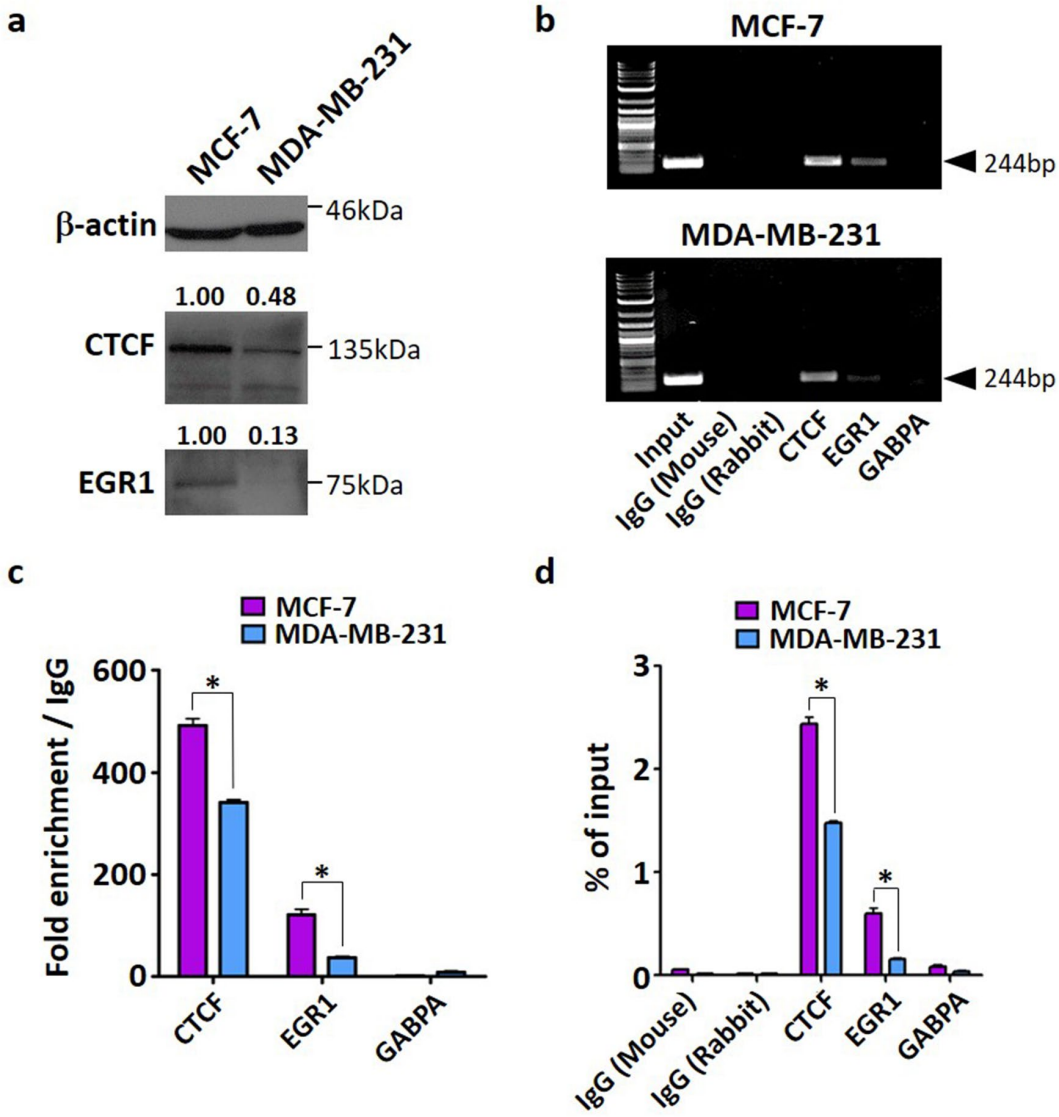
ChIP-qPCR of endogenous transcription factors binding to the *NME1* promoter in breast cancer cells. (**a**) MCF-7 and MDA-MB-231 cells were lysed and proteins were separated on 12% acrylamide gels. Quantification of protein bands was performed with ImageJ. (**b**) Protein-DNA complexes were pulled down by primary antibodies and protein G agarose beads, followed by qPCR. DNA gel electrophoresis was performed to analyze the presence of a 244 bp promoter region (− 244 to − 1 bp). ‘Input’ is the chromatin collected before antibody incubation. IgG (Mouse) and IgG (Rabbit) serve as antibody isotype controls. (**c**) Fold enrichment was calculated as fold change of Ct over corresponding IgG isotype controls. (**d**) Percentage of Input was calculated using Ct value of Input that represents 1% of starting chromatin. Data represent the mean and standard deviation of three independent trials. *p < 0.05.

**Figure 5:**
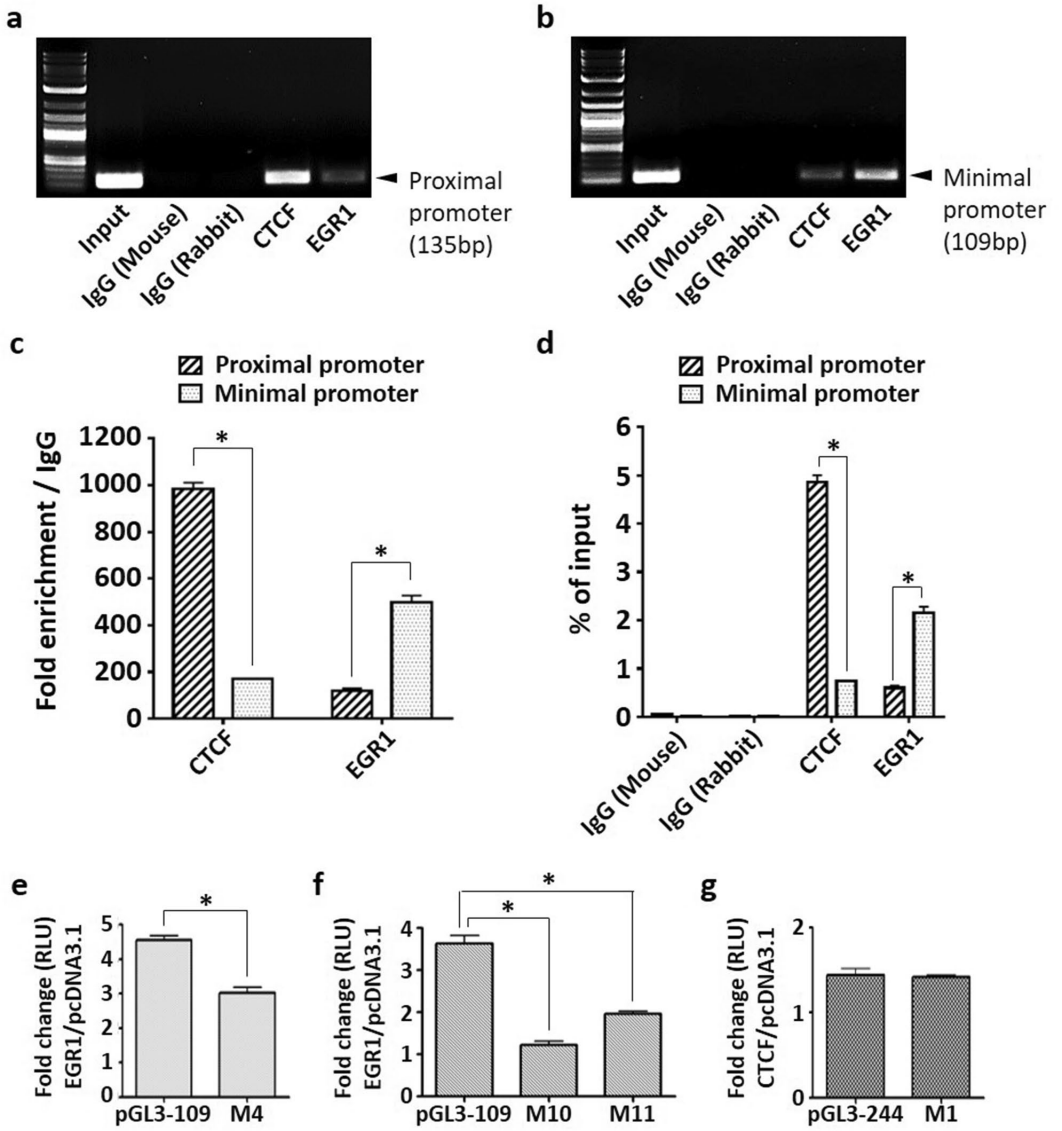
Binding of EGR1 to the *NME1* proximal and minimal promoters in MCF-7 cells. Protein-DNA complexes were pulled down by primary antibodies and protein G agarose beads, followed by qPCR. DNA gel electrophoresis was performed to analyze the presence of (**a**) the 136 bp proximal promoter (− 244 to − 109 bp) and (**b**) the 109 bp minimal promoter (− 109 to − 1 bp). ‘Input’ is the chromatin collected before antibody incubation. IgG (Mouse) and IgG (Rabbit) serve as antibody isotype controls. Data are shown as a representative experiment from three independent experiments. (**c**) Fold enrichment was calculated as fold change of Ct over corresponding IgG isotype controls. (**d**) Percentage of Input was calculated using Ct value of Input that represents 1% of starting chromatin. (**e**,**f**,**g**) MDA-MB-231 cells were transiently transfected with CTCF or EGR1 and promoter mutants. Cells were lysed 48 h after transfection and luciferase assays were performed using the Dual-Luciferase Reporter Assay System (Promega). Fold change was calculated as the increase in promoter activity by EGR1 or CTCF relative to pcDNA3.1 control. Data represent the mean and standard deviation of three independent trials. *p < 0.05.

It was previously shown that the endogenous expression of Nm23-H1 in highly aggressive MDA-MB-231 breast cancer cells is substantially lower than that of the less metastatic MCF-7 breast cancer cells (Fig. 1a), which may explain their distinct metastatic phenotype. As transcriptional activators of Nm23-H1, the endogenous expression of CTCF and EGR1 was also lower in MDA-MB-231 cells as compared to MCF-7 cells (Fig. 4a). To determine whether MCF-7 cells utilize CTCF and EGR1 to maintain Nm23-H1 expression and assume a less invasive phenotype, ChIP assays were performed in untransfected MCF-7 and MDA-MB-231 cells. Protein-DNA complexes were probed with primary antibodies for CTCF and EGR1, followed by qPCR to quantify the number of protein-bound promoters. The relative amount of promoter binding to CTCF and EGR1 was significantly higher in MCF-7 cells as compared to MDA-MB-231 cells as visualized by gel electrophoresis (Fig. 4b) and quantified as fold change to IgG control (Fig. 4c) and percentage of chromatin (Fig. 4d). Promoter binding to GABPA was absent in both cell lines, consistent with previous ChIP assays in transfected MDA-MB-231 cells. The binding of EGR1 was further analyzed separately at the proximal (− 244 to − 109 bp) and minimal (− 109 to − 1 bp) promoter, as algorithms predicted that binding sites 4, 10, and 11, may be responsive to EGR1 (Fig. 2a). Interestingly, EGR1 binding was significantly increased at the minimal promoter as compared to the proximal promoter (Fig. 5a–d). In contrast, CTCF binding was more prominent at the proximal promoter but negligible at the minimal promoter, consistent with a potential binding site for CTCF in the proximal promoter.

To further confirm the direct binding of CTCF and EGR1 to their specific binding sites, we checked whether promoter stimulation was lost when binding site mutants were expressed. MDA-MB-231 cells were transiently transfected with wild-type or mutant promoters, together with CTCF or EGR. Forced expression of EGR1 diminished the upregulation of wild-type promoter activity when the proximal promoter mutant M4 was expressed (Fig. 5e), and almost completely ablated when the minimal promoter mutants M10 or M11 mutants were expressed (Fig. 5f). However, the promoter upregulation by CTCF was not decreased in cells expressing the M1 mutant (Fig. 5g), which disrupts the binding site for CTCF according to the in silico screening. Nonetheless, sufficient evidence suggests that CTCF interacts with the Nm23-H1 promoter to induce its transcription. Collectively, these data indicate that CTCF and EGR1 act as transcription factors to drive Nm23-H1 expression in less metastatic breast cancer cells, whereas their lower expression in aggressive breast cancers may contribute to reduced levels of Nm23-H1. EGR1 binding was also more enriched at the minimal promoter as compared to the proximal promoter region of *NME1*.

### CTCF and EGR1 suppress cell migration of invasive breast cancer cells

**Figure 6:**
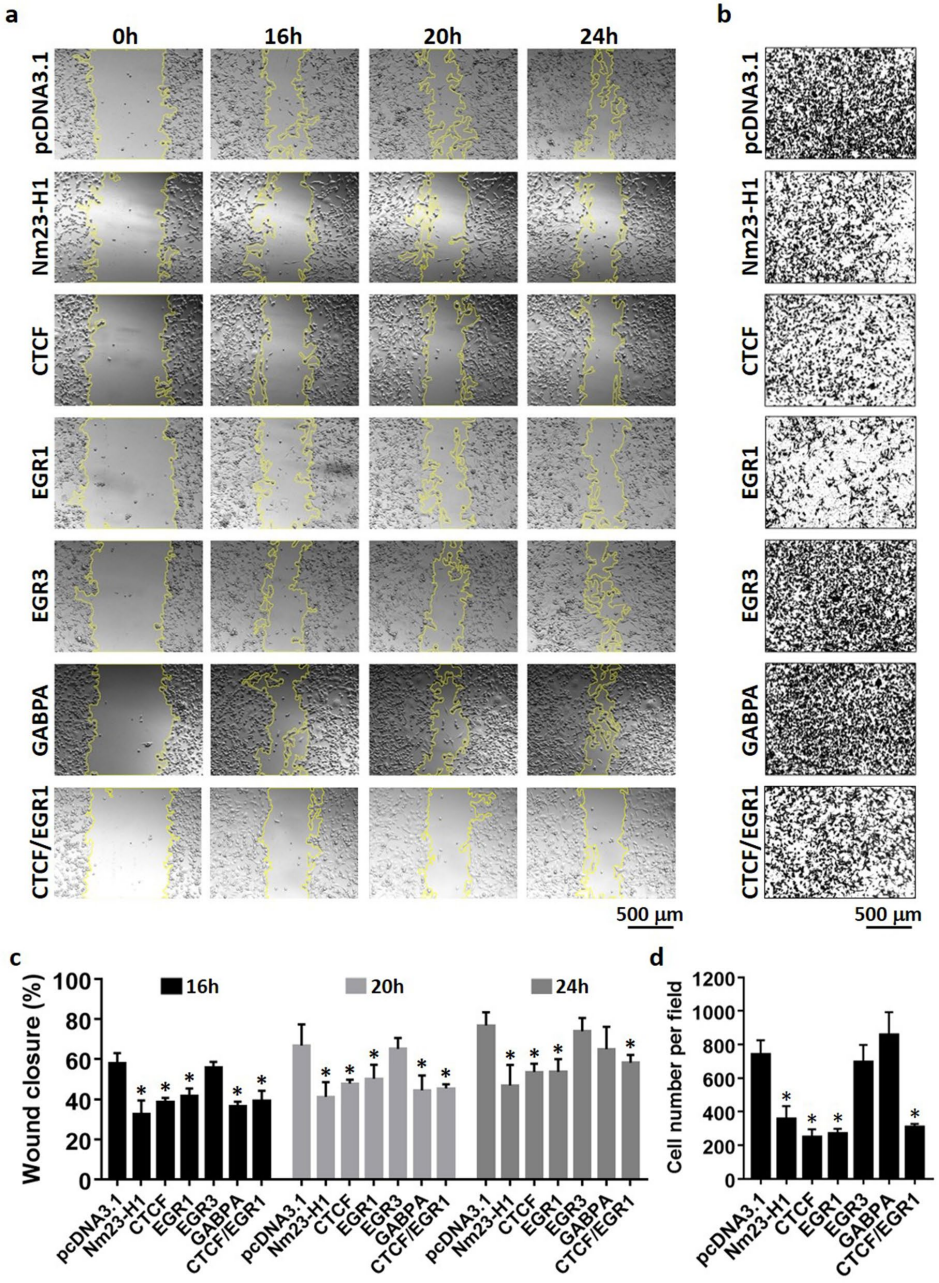
Cell migration of MDA-MB-231 cells expressing transcription factors. (**a**,**c**) Transiently transfected cells were grown to full confluency and scratched with a yellow pipette tip. Photos were taken at different time points with a microscope at ×10 magnification. Cell migration was quantified as percentage of wound closure using ImageJ and MRI Wound Healing Tool plugin. (**b**,**d**) Transiently transfected cells were seeded into the upper chamber of Transwell inserts and allowed for migration for 16 h. Photos were taken with a microscope at ×10 magnification and the number of migrated cells was quantified using ImageJ. Data represent the mean and standard deviation of three independent trials. *Significance with pcDNA3.1, p < 0.05.

**Figure 7:**
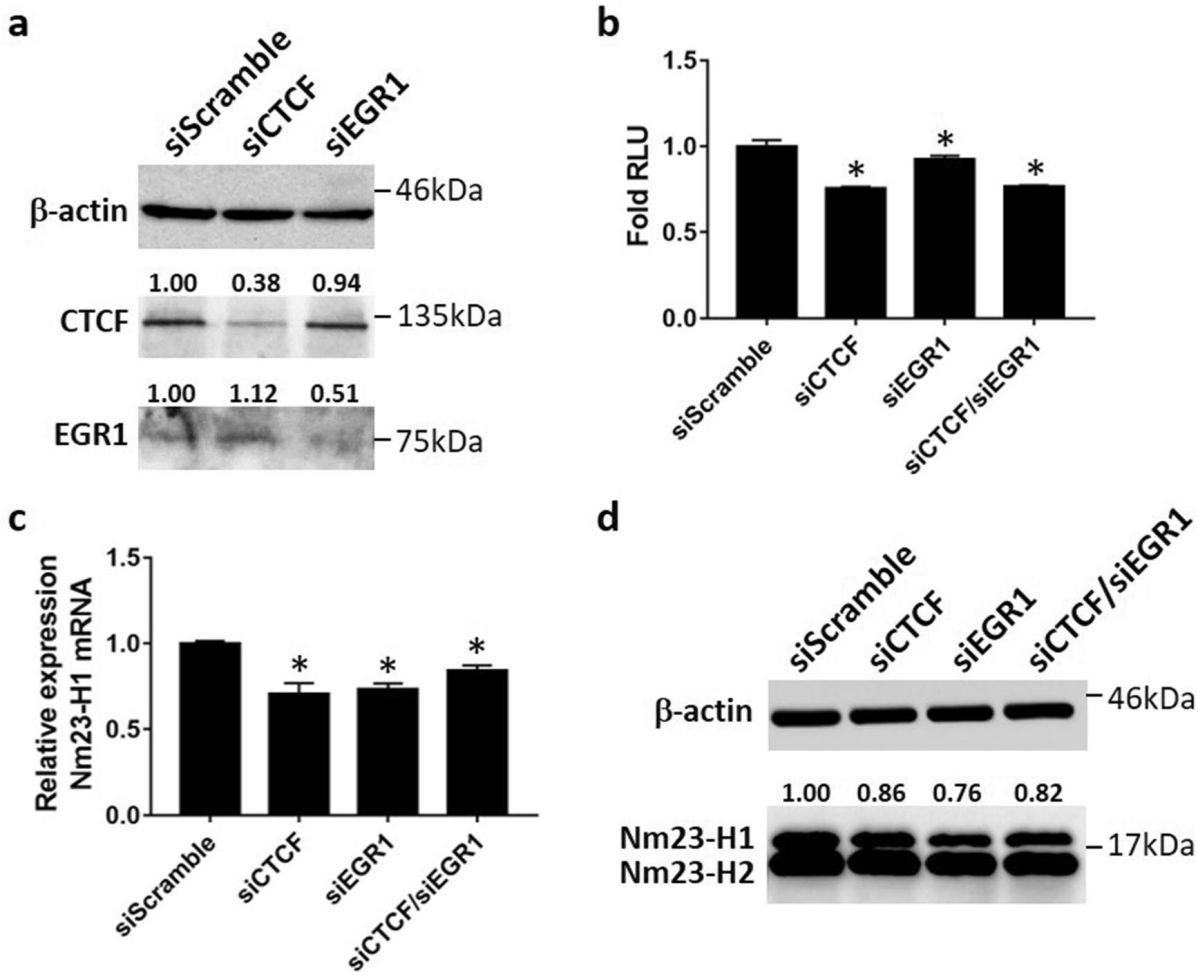
siRNA knockdown of CTCF and EGR1 in MCF-7 cells. (**a**,**d**) Untransfected MCF-7 cells or MCF-7 cells transiently transfected with siCTCF, siEGR1, or siScramble, were lysed 48 h after transfection. Proteins were separated on 12% polyacrylamide gels and immunoblots were probed with corresponding antibodies. Quantification of Nm23-H1 bands was performed with ImageJ. Data are shown as a representative experiment from three independent trials. (**b**) Luciferase assays were performed in transiently transfected cells using the Dual-Luciferase Reporter System (Promega) *Significant inhibition as compared to siScramble, p < 0.05. (**c**) RNA extraction was performed in transiently transfected MCF-7 cell, followed by cDNA synthesis and qPCR. *Significance with siScramble, p < 0.05. Data represent the mean and standard deviation of three independent trials.

**Figure 8:**
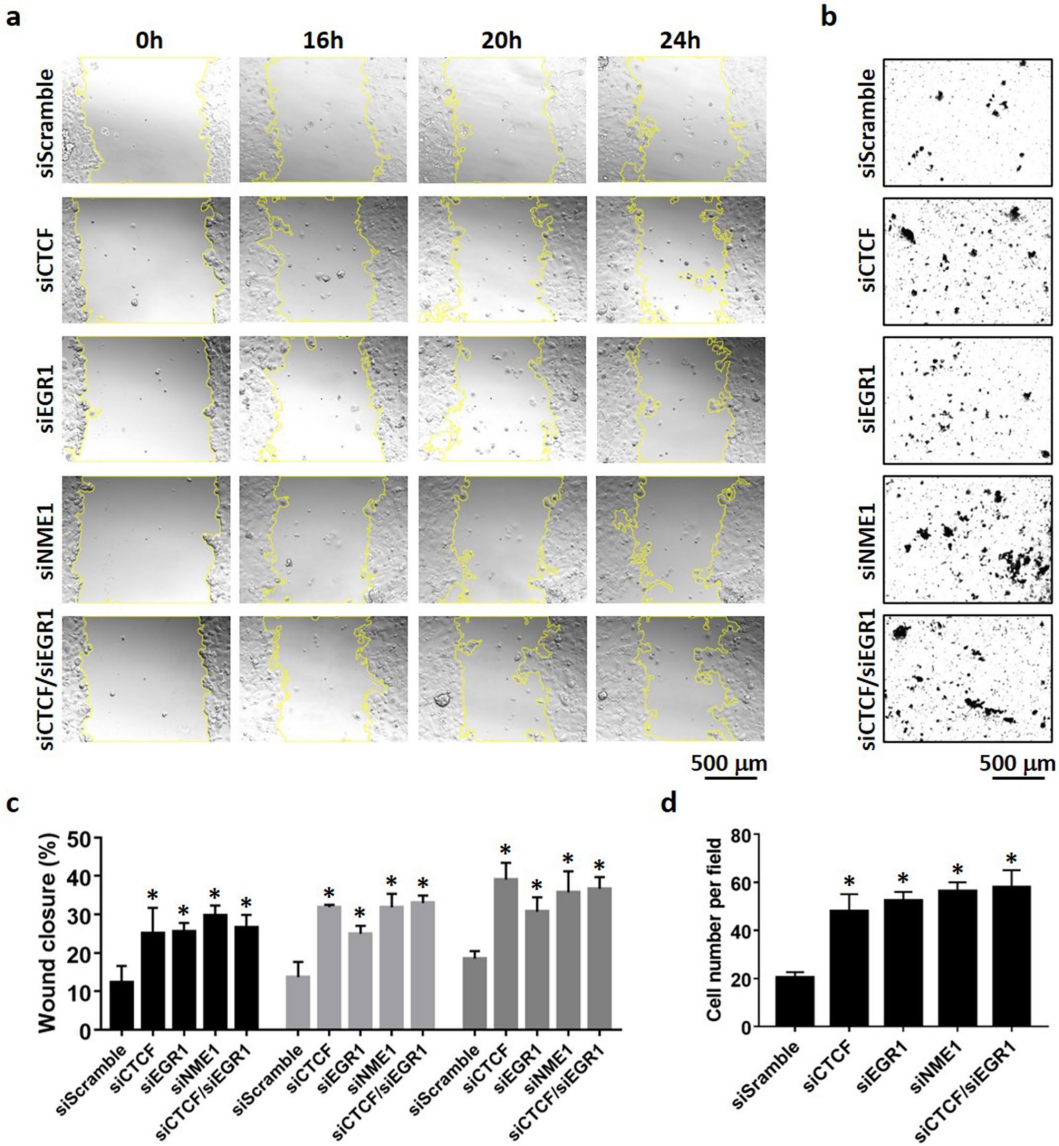
Cell migration of MCF-7 cells upon knockdown of CTCF and EGR1. (**a**,**c**) Transiently transfected cells were grown to full confluency and scratched with a yellow pipette tip. Photos were taken at different time points with a microscope at ×10 magnification. Cell migration was quantified as percentage of wound closure using ImageJ and MRI Wound Healing Tool plugin. (**b**,**d**) Transiently transfected cells were seeded into the upper chamber of Transwell inserts and allowed for migration for 48 h. Photos were taken with a microscope at ×10 magnification and the number of migrated cells was quantified using ImageJ. Data represent the mean and standard deviation of three independent trials. *Significance with siScramble, p < 0.05.

**Figure 9:**
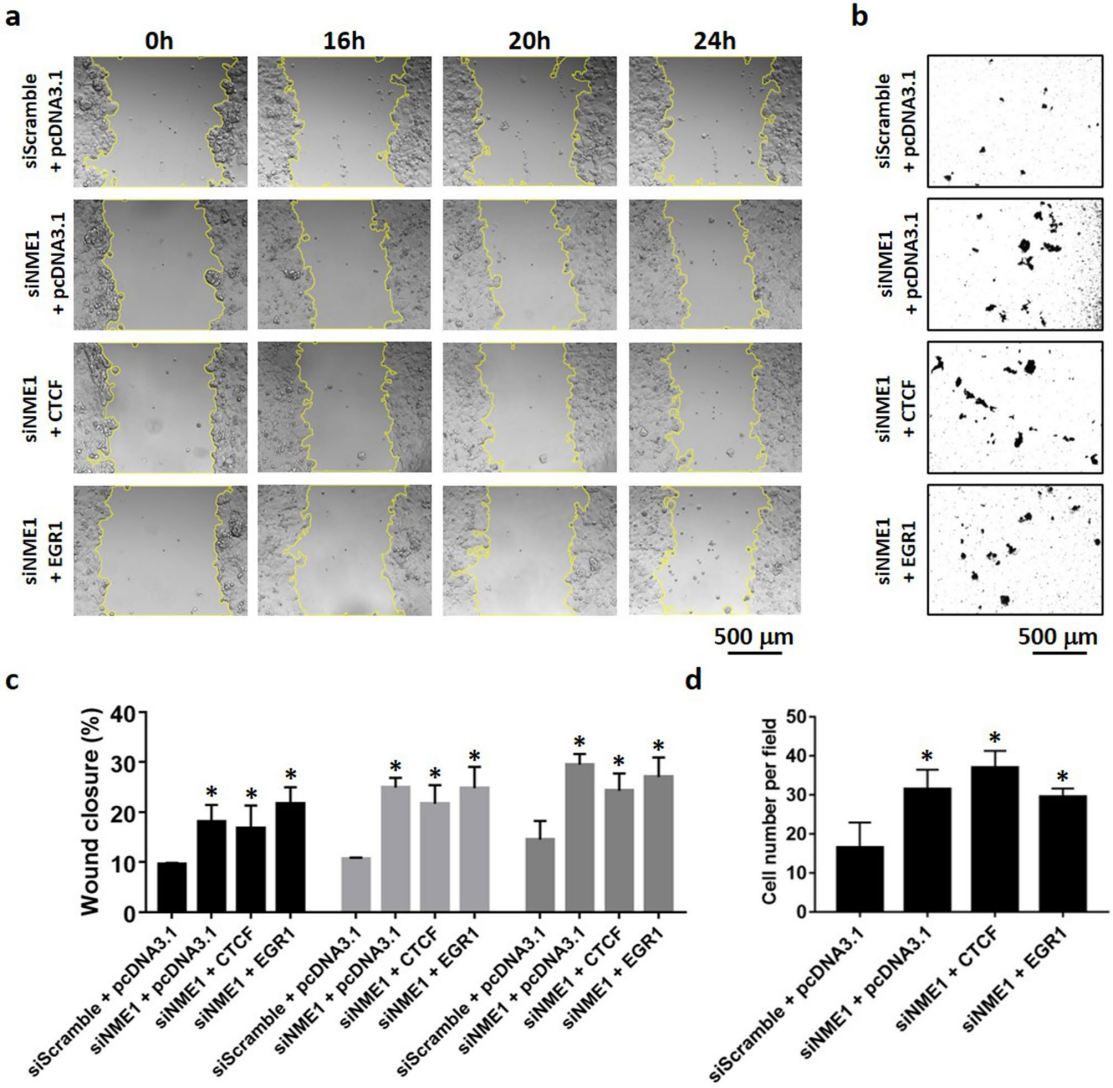
Cell migration of MCF-7 cells upon Nm23-H1 knockdown and CTCF or EGR1 overexpression. (**a**,**c**) Transiently transfected cells were grown to full confluency and scratched with a yellow pipette tip. Photos were taken at different time points with a microscope at ×10 magnification. Cell migration was quantified as percentage of wound closure using ImageJ and MRI Wound Healing Tool plugin. (**b**,**d**) Transiently transfected cells were seeded into the upper chamber of Transwell inserts and allowed for migration for 48 h. Photos were taken with a microscope at ×10 magnification and the number of migrated cells was quantified using ImageJ. Data represent the mean and standard deviation of three independent trials. *Significance with siScramble + pcDNA3.1, p < 0.05.

As CTCF and EGR1 have the ability to elevate Nm23-H1 levels, we hypothesized that CTCF and EGR1 may alter the aggressive phenotype of breast cancer cells through transcriptional control of *NME1*. To test this hypothesis, the migratory ability of transiently transfected MDA-MB-231 cells was examined by wound healing and transwell migration assays. MDA-MB-231 cells transiently expressing Nm23-H1 (Supplementary Fig. S4) significantly decreased wound closure as compared to pcDNA3.1 vector control at 16 h, 20 h, and 24 h time points (Fig. 6a,c), confirming its role as a metastasis suppressor in breast cancer cells. Forced expression of CTCF and EGR1 also suppressed cell migration at all time points, potentially caused by their ability to upregulate Nm23-H1. In contrast, EGR3 was found to associate with the Nm23-H1 promoter without enhancing Nm23-H1 protein levels (Fig. 3), which is reflected by its inability to abolish cell migration. Interestingly, GABPA significantly suppressed cell migration at earlier time points, whereas the difference in wound closure becomes insignificant at 24 h. Similar findings were observed in transwell migration assays, in which MDA-MB-231 cells were seeded into the top chamber of transwell inserts and allowed for migration through the membrane. Nm23-H1, CTCF, and EGR1 expression in MDA-MB-231 cells significantly decreased cell migration, whereas EGR3 and GABPA-expressing cells show similar number of migrated cells as compared to vector control (Fig. 6b,d). Double expression of CTCF and EGR1 inhibited cell migration at significant levels similar to cells expressing either transcription factor alone (Fig. 6a–d). To further demonstrate that CTCF and EGR1 are capable of regulating Nm23-H1 expression, siRNA-mediated knockdown of CTCF and EGR1 were performed in MCF-7 cells (Fig. 7a). Successful knockdown of CTCF or EGR1 significantly decreased *NME1* promoter activity (Fig. 7b), transcript levels (Fig. 7c) and protein expression (Fig. 7d), whereas the double knockdown (siCTCF/siEGR1) did not augment the inhibitory effect. Consistent with these findings, MCF-7 cells displayed enhanced migratory capabilities upon knockdown of CTCF or EGR1, as demonstrated by wound healing (Fig. 8a,c) and transwell migration assays (Fig. 8b,d). As a positive control, the knockdown of Nm23-H1 was also included in cell migration assays and caused efficient downregulation of Nm23-H1 transcript and protein levels (Supplementary Fig. S5). Forced expression of CTCF or EGR1 in siNME1-transfected MCF-7 cells did not reverse the invasive phenotype (Fig. 9a–d), indicating that CTCF and EGR1 inhibit cell migration mainly through the induction of Nm23-H1 expression. Collectively, these results indicate that breast cancer cells may utilize CTCF and EGR1 to drive Nm23-H1 expression and suppress the invasive phenotype, whereas the downregulation of these proteins in aggressive breast cancers potentially contribute to cancer metastasis.

## Discussion

Aberrant expression of Nm23-H1 is a common signature of breast cancers contributing to unique aggressive phenotypes. Previous studies that attempted to identify regulatory mechanisms of Nm23-H1 expression have proposed the involvement of several transcription factors. In the present study, we sought for transcription factors that are responsible for maintaining Nm23-H1 expression at sufficient levels to control the metastatic process. The less metastatic MCF-7 and highly aggressive MDA-MB-231 breast cancer cell lines represent phenotypic models that exhibit high or low expression of Nm23-H1 protein, respectively. Nm23-H1 protein and mRNA levels were abrogated in MDA-MB-231 cells as compared to MCF-7 cells, suggesting that reduced *NME1* transcription is a probable cause of downregulation. Based on promoter truncation studies in breast cancer cell lines, a proximal promoter region (from − 244 to − 109 bp) and a minimal promoter region (from − 109 to − 1 bp) were apparently responsible for driving Nm23-H1 expression. Other regions more upstream of the proximal promoter were presumed less important in defining transcriptional activation and may harbor repressive elements that limit reporter activity. More importantly, the activity of promoter constructs correlated well with mRNA and protein levels in MDA-MB-231 and MCF-7 cells, suggesting that transcriptional control of *NME1* is a major contributor to the downregulation of Nm23-H1. Consistent with these findings, histone modification data extracted from ENCODE^44^ in the UCSC Genome Browser revealed that the proximal and minimal promoter regions are bordered by high contents of H3K27ac and H3K4me3 and a lower content of H3K4me1, emphasizing that transcription factors may access these regions of the promoter to exert their regulatory functions.

In silico screenings and mutagenesis studies generated a list of potential candidates binding to the *NME1* promoter. The binding of transcriptional repressors at the *NME1* promoter, such as the thyroid hormone receptor in hepatoma cell lines^45^, is unlikely responsible for the low expression of Nm23-H1 in MDA-MB-231 cells, which were incapable of showing enhanced promoter activity when transcription factor binding sites were disrupted. It was demonstrated that binding sites 3–6 significantly contribute to *NME1* promoter activity in MCF-7 cells, whereas only binding site 6 stimulated the *NME1* promoter in MDA-MB-231 cells, in line with reduced transcriptional activation of *NME1* in these cells. CTCF and EGR1 were among the most promising transcription factors because of their ability to induce *NME1* promoter activity, transcript levels, and protein levels in MDA-MB-231 cells, as well as interact with the *NME1* promoter in MCF-7 cells. In addition to the transcription factors analyzed in this study, the estrogen receptor-α also possesses transactivation potential to induce the *NME1* promoter in several breast cancer cell lines, which may further contribute to the transcriptional activation of Nm23-H1^46^.

**Figure 10:**
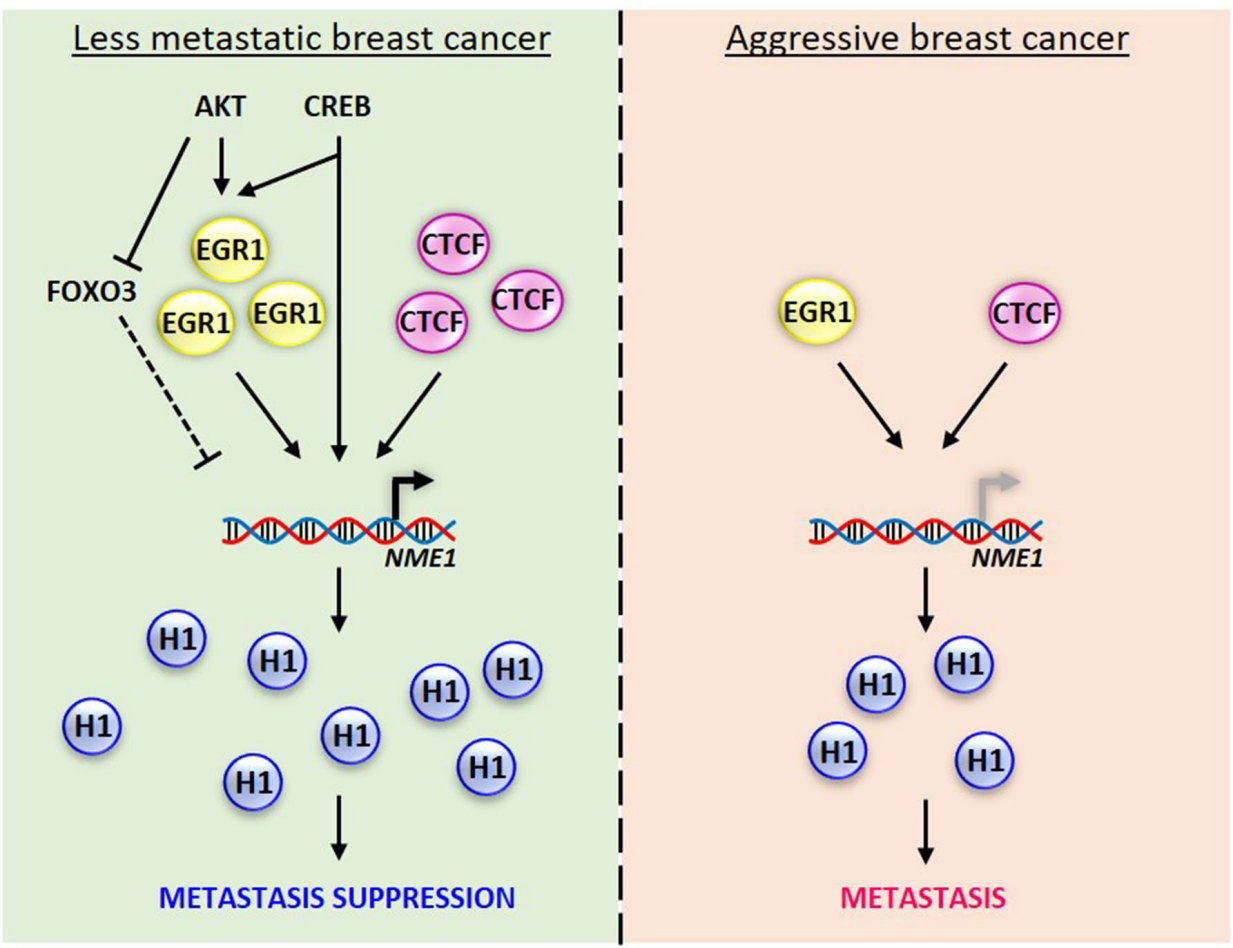
Proposed model of pathways regulating Nm23-H1 expression. CTCF and EGR1 are recruited to the promoter of *NME1* to activate transcription, potentially contributing to metastasis suppression. The expression of Nm23-H1 can be further regulated by the action of AKT, which activates EGR1 and inhibits FOXO3, a negative regulator of *NME1* transcription. In addition, CRE regions in the promoters of *EGR1* and *NME1* may be targeted by CREB to drive the expression of Nm23-H1. The low expression of CTCF and EGR1 in aggressive breast cancer cells may contribute to decreased Nm23-H1 protein levels and metastasis.

Although the role of CTCF and EGR1 in breast cancer cell proliferation are more well-established as compared to their functions in metastasis, this study supports metastasis suppressor roles for CTCF and EGR1 in breast cancer by acting as positive regulators of *NME1* transcription (Fig. 10). In particular, the dysfunction of CTCF caused by the K334E mutation in breast cancer has been associated with the onset of the disease^47^, which could affect its ability to bind to the promoter of genes related to cell proliferation including MYC, PLK, and p19ARF^48^. EGR1 has been implicated in the inhibition of cell cycle progression in breast cancer through transcriptional repression of cyclin D1, D2, and D3^33,34^. In addition, the transactivation of PTEN by EGR1 results in the inhibition of PI3K/AKT signaling, in which AKT activates EGR1 by phosphorylation and completes a negative feedback loop^49^. This pathway also supports the positive correlation between AKT and Nm23-H1 protein levels in lung cancer models^50^, which was demonstrated to rely on the inhibition of FOXO3^24^ (Fig. 10). The ability of EGR1 to drive Nm23-H1 expression may also augment the upregulation of Nm23-H1 induced by CREB^25^ (Fig. 10), which can bind to CRE regions in the *EGR1* and *NME1* promoters to activate transcription^51^. The endogenous expression of CTCF and EGR1 were correlated with Nm23-H1 expression in MCF-7 cells, while the ability of CTCF and EGR1 to reduce cell motility was demonstrated in wound healing and transwell migration assays using MDA-MB-231 cells. Moreover, downregulation of EGR1 and CTCF is frequently observed in invasive breast tumors^32,33^, similar to the downregulation of Nm23-H1 in MDA-MB-231 cells. These findings indicate that *NME1* transcription is possibly maintained at sufficient levels by CTCF and EGR1 in less invasive breast cancer cells to suppress the aggressive phenotype. However, the binding of CTCF to the predicted binding site in the *NME1* promoter could not be verified, which might be caused by the multivalent DNA binding recognition of CTCF zinc fingers^52^. In contrast, multiple binding sites were observed for EGR1, whose binding was more enriched at the minimal promoter containing two EGR1 recognition sites as compared to the proximal promoter region displaying a single site for interaction. The presence of multiple binding sites for EGR1 emphasizes the biological importance of this interaction and may represent a robust pathway to control Nm23-H1 expression and cancer metastasis. Additional genes involved in cell migration and invasion are likely regulated by these transcription factors to induce the cellular phenotype. In fact, putative binding sites for both CTCF and EGR1 are found in the promoter of other metastasis suppressor genes including BRMS1, CDH1, KAI1, NDRG1, and RKIP according to GeneHancer^53^. The co-regulation of these genes may contribute to the metastasis suppressor functions of CTCF and EGR1.

Other transcription factors predicted by algorithms were proven incapable to induce Nm23-H1 protein expression transcriptionally. Although disruption of the M6 binding site caused the most significant reduction in reporter activity of the proximal promoter region, a bona fide regulator of Nm23-H1 expression could not be identified among ETS family members. For instance, GABPA robustly increased Nm23-H1 mRNA and protein levels, but significantly reduced promoter activity. Promoter binding was also absent in MCF-7 and MDA-MB-231 cells, indicating that GABPA may upregulate Nm23-H1 protein expression through other non-transcriptional mechanisms in breast cancer cells. In contrast, ELF5, KLF2, and PU.1 upregulated mRNA, protein, and promoter activity but were not detected in ChIP assays, suggesting that they may activate *NME1* transcription indirectly or bind to DNA sequences beyond the proximal and minimal promoter regions. The failure of transcription factor binding at the *NME1* promoter in ChIP assays was not caused by somatic mutations, which appear to be absent in the analyzed promoter region in breast cancer tissues according to the COSMIC database^18^. These results demonstrate that the induction of mRNA, protein, or promoter activity does not guarantee promoter binding and transcriptional activation, and vice versa. Nonetheless, this study has revealed significant elements in the *NME1* promoter responsible for gene expression. *NME1* can be transcriptionally regulated by a multitude of transcription factors, in particular by CTCF and EGR1, which potentially drive Nm23-H1 expression in breast cancer cells to promote a less metastatic phenotype. The discovery of transcriptional mechanisms may provide novel strategies for therapeutic intervention that aims to control Nm23-H1 expression and the metastatic disease.

## Methods

### Reagents

The cDNA of CTCF was a gift from Jesse Boehm & William Hahn & David Root (Addgene plasmid #81789). HA-tagged CK2α was a gift from David Litchfield (Addgene plasmid #27086). The cDNA of EGR1 was a gift from Shinya Yamanaka (Addgene plasmid #52724). The cDNA of PU.1 was a gift from George Daley (Addgene plasmid #97039). Plasmids for ELF5 and HA-tagged GABPA were purchased from Sino Biological Inc. (Beijing, China). Breast cancer cell lines MCF-7 and MDA-MB-231 were purchased from American Type Culture Collection (ATCC). T47D and MDA-MB-468 cell lines were a kind gift from Prof. Ava Kwong (University of Hong Kong, Hong Kong). Custom primers, TRIzol reagent, SuperScript III First-Strand Synthesis Kit, cell culture reagents, ChIP reagents, Lipofectamine 2000 and Lipofectamine RNAiMAX, were purchased from Invitrogen (Carlsbad, USA). LightCycler 480 SYBR Green I Master Kit was obtained from Roche (Penzberg, Germany). Dual-Luciferase Reporter Assay System was purchased from Promega (Madison, USA). Costar Transwell permeable supports were purchased from Corning Incorporated, Life Sciences (New York, USA). Anti-HA (12CA5) antibody was purchased from Roche (Penzberg, Germany). Antibodies for EGR1 (#4153), IgG-Mouse (#5415), IgG-Rabbit (#3900), and Nm23-H1/H2 (#3345), were purchased from Cell Signaling Technology (Danvers, USA). GABPA (sc-28312) antibody was purchased from Santa Cruz Biotechnology (Dallas, USA). CTCF (07-729) and β-actin (A1978) antibodies were obtained from Merck Millipore (Burlington, USA). Secondary antibodies were obtained from GE Healthcare (Chicago, USA). Silencer select siRNAs for CTCF (s20966), EGR1 (s4538), Nm23-H1 (s9588), and scramble (4390843), were purchased from Ambion (Life Technologies).

### Cell culture and transfection

MDA-MB-231 cells were cultured in DMEM supplemented with 10% FBS, 0.5% Pen/Strep at 37 °C, 5% CO_2_. MCF-7 cells were cultured in RPMI-1640 with 10% FBS, 0.5% Pen/Strep at 37 °C, 5% CO_2_. For transient transfection of MCF-7 and MDA-MB-231 cell lines, cells were seeded 1 day prior to transfection in 6-well plates to reach a confluence of 70–80%. 1 μg of DNA with Lipofectamine 2000, or 15 pmol siRNA with Lipofectamine RNAiMAX, was used for transfection according to manufacturer’s protocol in antibiotics/serum-free OPTI-MEM. 4 h after transfection, cells were supplemented with 10% FBS and 0.5% Pen/Strep.

### Extraction of genomic DNA

HEK293 cells were grown to confluence and lysed with TRIzol (Invitrogen). Phase separation was performed according to manufacturer’s protocol. After removing the aqueous phase, the interphase and organic phase were centrifuged for 5 min, 4 °C, 12,000×*g*. Back extraction buffer (4 M guanidine thiocyanate, 50 mM NaCl, 1 M Tris) was added and mixed for 3 min by inversion. Samples were centrifuged for 30 min, 12,000×*g* at room temperature and the upper aqueous phase was transferred to a clean tube. DNA precipitation was performed with isopropanol for 5 min at room temperature. The DNA pellet was washed with 70% ethanol and centrifuged for 15 min, 4 °C, 12,000×*g*. Quality of genomic DNA dissolved in TE buffer was determined by measuring the A260/A280 ratio using NanoDrop 2000 (Thermo Scientific).

### Cloning of DNA constructs

The promoter region of NME1, cDNA of ELF1 and OLF1, were cloned from HEK293 genomic DNA with specific primers and ligated into suitable vectors (Supplementary Table S1). PCR was performed using isolated genomic DNA as template and gene-specific primers containing restriction sites for cloning into plasmids. Plasmids were transformed in competent DH5α *E. coli* cells for DNA purification. Plasmid sequences were verified by Sanger sequencing (BGI).

### Western blotting

Western blot was performed as described previously^25^. In brief, cells were lysed and cell debris was removed by centrifugation at top speed for 5 min at 4 °C. Proteins were resolved by 12% or 15% SDS-PAGE and transferred to nitrocellulose membranes. The membranes were blocked and sequentially incubated with corresponding primary and secondary antibodies with washing steps in between. Immunoblots were visualized by chemiluminescence using WesternBright ECL (Advansta). Unprocessed versions of original immunoblots can be found in Supplementary Figs. S6, S7, and S8. Several blots were cut prior to antibody hybridization for the simultaneous detection of different target proteins in the same sample.

### Quantitative PCR

Total RNA was extracted from cells using TRIzol reagent (Invitrogen) according to manufacturer’s protocol. RNA samples were treated with DNase I (Invitrogen) for 30 min at 37 °C and re-extracted using TRIzol. RNA quality and quantity were determined by Nanodrop 2000 (Thermo Scientific). cDNA synthesis was performed with SuperScript III First-Strand Synthesis Kit (Invitrogen) according to manufacturer’s protocol. Synthesized cDNAs were mixed with LightCycler 480 SYBR Green I Master mix (Roche) and forward and reverse primers as listed in Supplementary Table S2. The fold change was calculated by the difference in threshold cycles (Ct) between test and control samples.

### Luciferase reporter gene assay

Confluent cells were transfected with NME1 promoter constructs and the internal control vector pRL-TK (10:1 ratio) to adjust for transfection efficiencies of different cell lines. Cells were lysed with Passive Lysis Buffer (Promega) 48 h after transfection and agitated for 30 min at 4 °C. Samples were centrifuged at top speed for 5 min and cell debris was removed. Protein samples were transferred to a white 96-well plate and the luciferase activities were measured using the Dual-Luciferase Reporter Assay System (Roche) in a SpectraMax L microplate reader (Molecular Devices LLC). *Firefly* luciferase activities were normalized to *renilla* luciferase activities and expressed as relative light units (RLU).

### ChIP assay

After transfection, proteins and DNA were crosslinked with formaldehyde and rotated gently at room temperature for 10 min. Glycine was added to the mixture and incubated with gentle shaking for 5 min. Cells were washed twice with ice-cold PBS and collected in PBS, followed by centrifugation for 5 min, 4 °C, 1000×*g*. The supernatant was aspirated and the pellet was resuspended in ChIP lysis buffer (50 mM HEPES KOH pH 7.5, 140 mM NaCl, 1 mM EDTA pH 8, 1% Triton X-100, 0.1% sodium deoxycholate, 0.1% SDS, supplemented with protease inhibitors). The chromatin was sheared by sonication and centrifuged for 10 min, 4 °C, 8000×*g*. 10 μg DNA was used per immunoprecipitation and diluted 1:10 in IP dilution buffer (1% Triton X-100, 2 mM EDTA pH 8, 20 mM Tris–HCl pH 8, 150 mM NaCl, supplemented with protease inhibitors). 10 μg of corresponding antibodies was added to the sample and rotated for 1 h at 4 °C. Protein G agarose beads were added to the sample and rotated overnight at 4 °C. Samples were centrifuged for 1 min at 2000×*g* and the beads were washed 3 times in washing buffer (0.1% SDS, 1% Triton X-100, 2 mM EDTA pH 8, 20 mM Tris–HCl pH 8, 150 mM NaCl) and once in final washing buffer (0.1% SDS, 1% Triton X-100, 2 mM EDTA pH 8, 20 mM Tris–HCl pH 8, 500 mM NaCl). Elution was performed by the addition of elution buffer and crosslinking was reversed by addition of proteinase K (20 mg/mL; 60 °C for 1 h). The DNA was purified by PCI extraction and directly used for PCR. Unprocessed versions of original agarose gels can be found in Supplementary Fig. S9.

### Wound healing assay

Wound healing assay was performed as previously described^54^. In brief, wounds were applied in transiently transfected MDA-MB-231 or MCF-7 cells using a yellow pipette tip and cells were washed with PBS. Cells were cultured in complete growth medium with or without drugs and photos were taken at indicated time points using a microscope with 10 × magnification. Cell migration was quantified as percentage of wound closure using ImageJ and MRI Wound Healing Tool plugin.

### Transwell migration assay

Transwell migration assay was performed as previously described^25^. Briefly, 7.5 × 10^4^ MDA-MB-231 cells or 1.0 × 10^5^ MCF-7 cells were seeded with serum-free medium into the upper chamber of 24-well Costar Transwell permeable supports with 8.0 μm pore size (Corning). The lower chamber of the well was filled with complete growth medium containing 10% FBS. Cells were cultured for 16 h (MDA-MB-231) or 48 h (MCF-7) and washed with PBS twice. Cells were fixed with 4% PFA for 15 min and washed twice with PBS. Staining was performed with 0.5% crystal violet for 15 min in the dark. Cells were washed twice with PBS and removed from the upper chamber of the insert using a cotton swab. Photos were taken with a microscope at 10 × magnification and the number of migrated cells was quantified using ImageJ.

### In silico screening of transcription factors and miRNA interactions

The software tools MatInspector^28^ and the TRANSFAC database^29^ were utilized to predict potential transcription factors binding to the Nm23-H1 promoter. Potential transcription factors were selected based on their algorithm scoring and consistent prediction in both software.

### Data analysis

Data was obtained from at least three independent experiments and presented as the mean with standard deviation. The significance was calculated using the student’s t-test; a p-value of less than 0.05 was considered as significant. Western blots were quantified using ImageJ software. All graphs and significance were plotted and calculated using GraphPad Prism software.

## Supplementary Information

Supplementary Information.
