# Supplementary material for: CTCF and EGR1 suppress breast cancer cell migration through transcriptional control of Nm23-H1
Source: Sci Rep. 2021 Jan 12;11:491. doi: 10.1038/s41598-020-79869-9 (PMC7804126; doi:10.1038/s41598-020-79869-9)
Supplement: Supplementary file 1 — Supplementary Information. [file 41598_2020_79869_MOESM1_ESM.pdf]

CTCF and EGR1 suppress breast cancer cell migration through transcriptional control of Nm23-H1

Ka Ming Wong[1], Jiaxing Song[1], Yung H. Wong[1,2]

[1]Division of Life Science and the Biotechnology Research Institute, Hong Kong University of Science and Technology, Clear Water Bay, Kowloon, Hong Kong.

[2]State Key Laboratory of Molecular Neuroscience, Hong Kong University of Science and Technology, Clear Water Bay, Kowloon, Hong Kong.

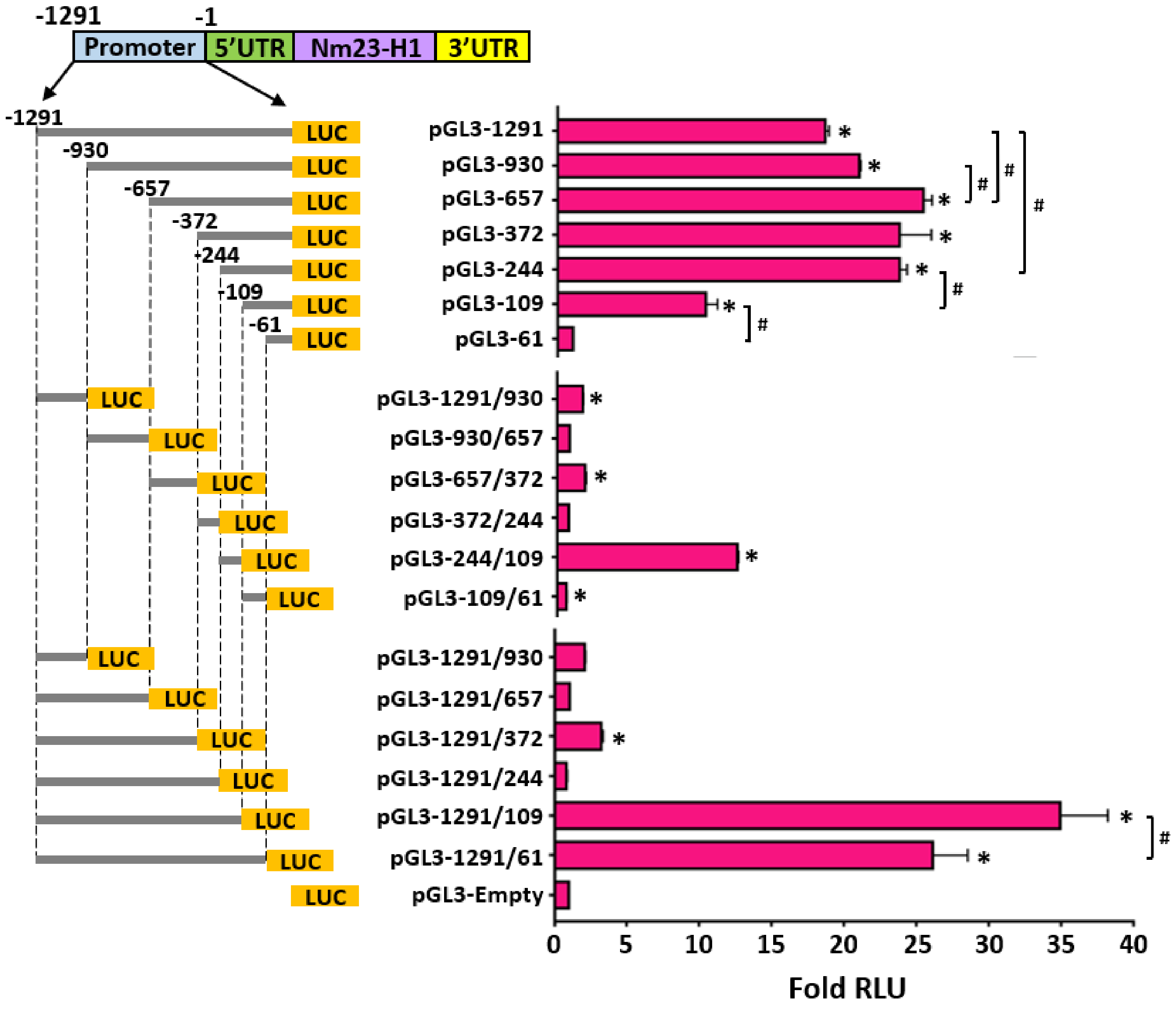

Supplementary Figure S1. Analysis of Nm23-H1 promoter constructs in HEK293 cells. HEK293 cells were transiently transfected with Nm23-H1 promoter constructs and pRL-TK control vector and lysed 48 hours after transfection. Luciferase activities were measured using the Dual-Luciferase Reporter Assay System (Promega). RLU values of reference construct pGL3-Empty: *Firefly* luciferase (1,128,152 ± 13,073), *Renilla* luciferase (3,427,559 ± 51,849). Data represent the mean and standard deviation of three independent trials. *, significance with pGL3-Empty, $p < 0.05$; #, $p < 0.05$.

Proximal promoter region (-244 bp to -109 bp)

```
         *************** *******    **** **** ****  ************* **    * *  * ****  *
  human  GTGTGAGCGCCACCTCTCGGGAAGCCAATTTGCTCGCGAACGAAGGAAGTGAGTCAGAGAACCCGGGGGTGGAGAGA
macaque  GTGTGAGCGCCACCTCTCGGGAAGCCAATTTGCTCGCGAACAAAGGAAGTGAGTCAGAGAACCCGGGGGTGGAGGGA
  mouse  GTGTGAGCGCCACCTGTCGGGAACTAGATTTTCTCGGGAACGGAGGAAGTGAGTCACAGGCAGCCGTAGGGGAGAAA

            ***   ** ** *  * *   **    ***** * ** * ************    *     ** *********
  human  AGAAAGCAAGCAGCTAACCGGAAAGGTCTGAAAAAGCTAGCGCCGGAAGTTAAACTGCAGAAAGTTGTTCCCTAGAA
macaque  AGAAAGCAAGCAGCTAACCGGAAAGGTCTGAAAAAGCTAGCGCCGGAAGTTAAACAGCAGAAAGTTGTTCCCTAGAA
  mouse  CAGAAGATGGCCGCCAGACAGGGGGGGTAAAAAAATCCAGTGTCGGAAGTTAAACACTGGGCGAGTGCTCCCTAGAA
```

Minimal promoter region (-109 bp to -1 bp)

```
         ****  ** ****** *** ****** ** *  ******* * ** *** ****** ** * * **** ***  *
  human  TGACTGCCTACTCCCAAGAGGAAGCGTGGGCGAGCGGTGTTCTGCAAAATGGGCTCTCCGGCAGCGGATCTGCGCAG
macaque  TGACTGCCTACTCCCAAGAGGAAGCGTGGGCGAGCGGTGTTCTGCAAAATGGGCTCTCCGGCAGCGGATCTGCGCGG
  mouse  TGACATCCGACTCCCCAGAAGAAGCGGGGACCGGCGGTGTACCGCTAAAAGGGCTCCCCTGAATCGGAACTGTCCGA

         **  ********
  human  AAGCGTTCCGTG
macaque  AAGCGTTCCGTG
  mouse  AAAGGTTCCGTG
```

Supplementary Figure S2. Cross-species alignment of *NME1* promoter region. The proximal and minimal promoter region of *NME1* were aligned to the macaque and mouse sequence using the ECR Browser [55] and ClustalW2 alignment tool [56]. Conserved bases in all three species are indicated by an asterisk.

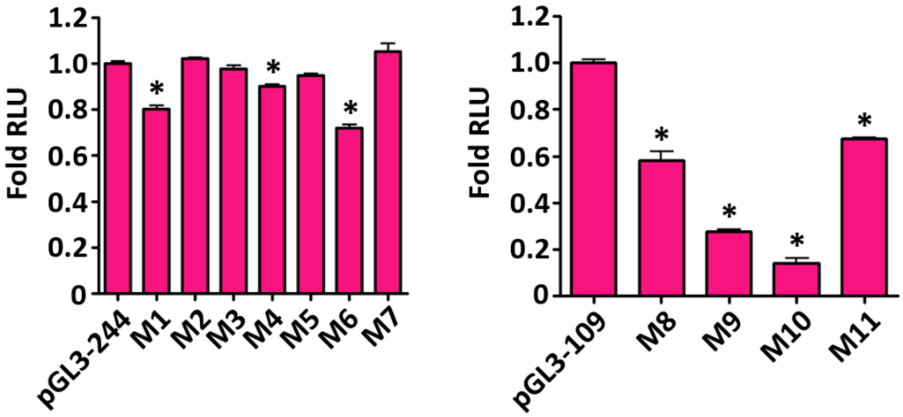

Supplementary Figure S3. Site-directed mutagenesis of transcription factor binding sites. Mutants of the proximal promoter (M1-M7) and minimal promoter (M8-M11) were transiently transfected in HEK293 cells and lysed 48 h after transfection. Luciferase assays were performed using the Dual-Luciferase Reporter Assay System (Promega). RLU values of reference constructs: pGL3-244, *Firefly* luciferase (31,948,369 ± 403,518), *Renilla* luciferase (3,908,541 ± 434,200); pGL3-109, *Firefly* luciferase (5,316,166 ± 29,785), *Renilla* luciferase (1,329,707 ± 91,446). Data represent the mean and standard deviation of three independent trials. *, significance with wild-type promoter, $p < 0.05$.

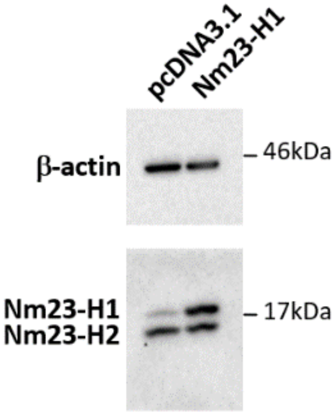

Supplementary Figure S4. Transient expression of Nm23-H1 in MDA-MB-231 cells. MDA-MB-231 cells were transiently transfected with Nm23-H1 or pcDNA3.1 control vector. Proteins were separated on 15% acrylamide gels and immunoblots were probed with corresponding antibodies. Data are shown as a representative experiment from three independent trials.

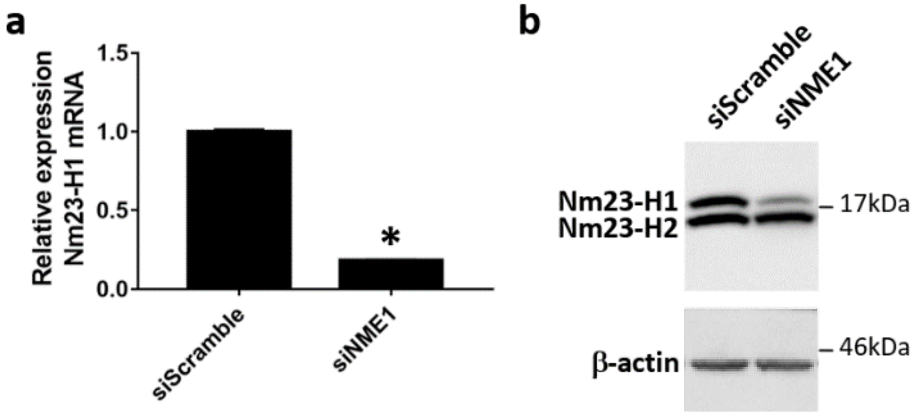

Supplementary Figure S5. Knockdown of Nm23-H1 by siRNA in MCF-7 cells. (**a**) MCF-7 cells were transiently transfected with siNME1 or siScramble control. RNA extraction was performed with TRIzol, followed by cDNA synthesis and qPCR. *, significance with siScramble, $p < 0.05$. Data represent the mean and standard deviation of three independent trials. (**b**) Proteins were separated on 15% acrylamide gels and immunoblots were probed with corresponding antibodies.

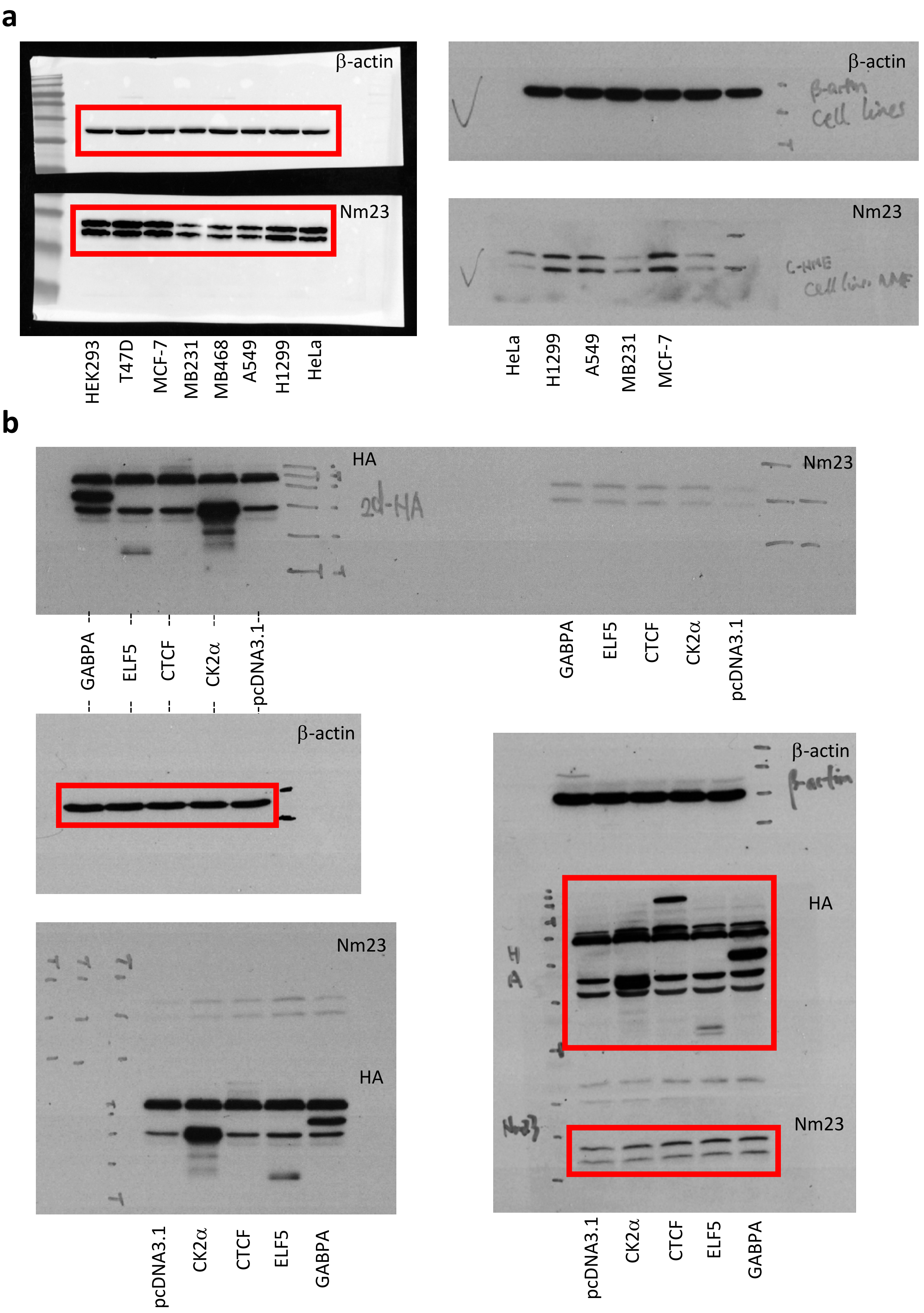
a
β-actin
Nm23
HEK293
T47D
MCF-7
MB231
MB468
A549
H1299
HeLa
β-actin
Nm23
HeLa
H1299
A549
MB231
MCF-7
b
HA
Nm23
GABPA
ELF5
CTCF
CK2α
pcDNA3.1
GABPA
ELF5
CTCF
CK2α
pcDNA3.1
β-actin
β-actin
HA
Nm23
HA
Nm23
pcDNA3.1
CK2α
CTCF
ELF5
GABPA
pcDNA3.1
CK2α
CTCF
ELF5
GABPA

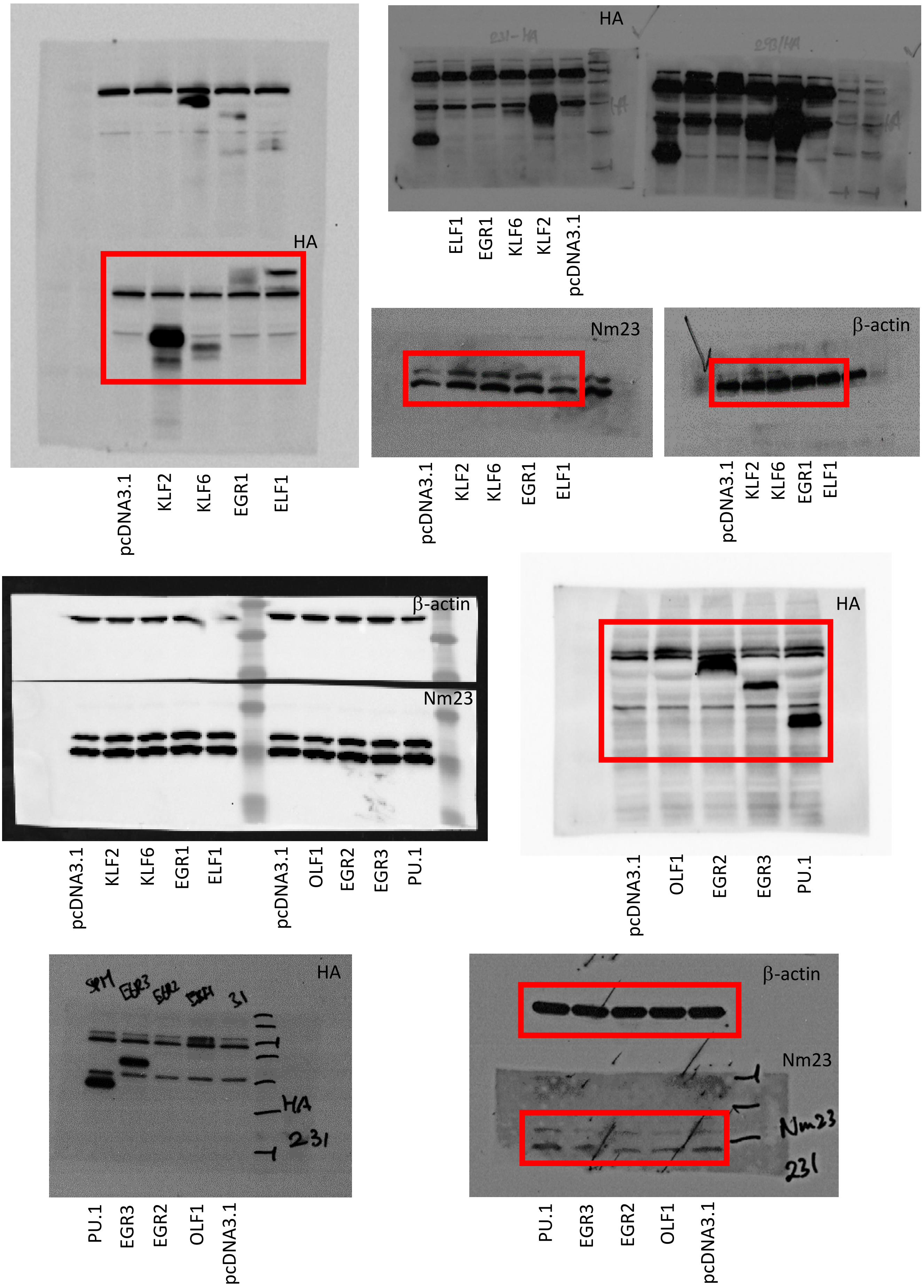

Supplementary Figure S6. Unprocessed original version of Western blots (1). (**a**) Analysis of Nm23-H1 protein expression in breast cancer cell lines, **Fig. 1a**. (**b**) Transcription factors regulating Nm23-H1 protein levels in MDA-MB-231 cells, **Fig. 3c**.

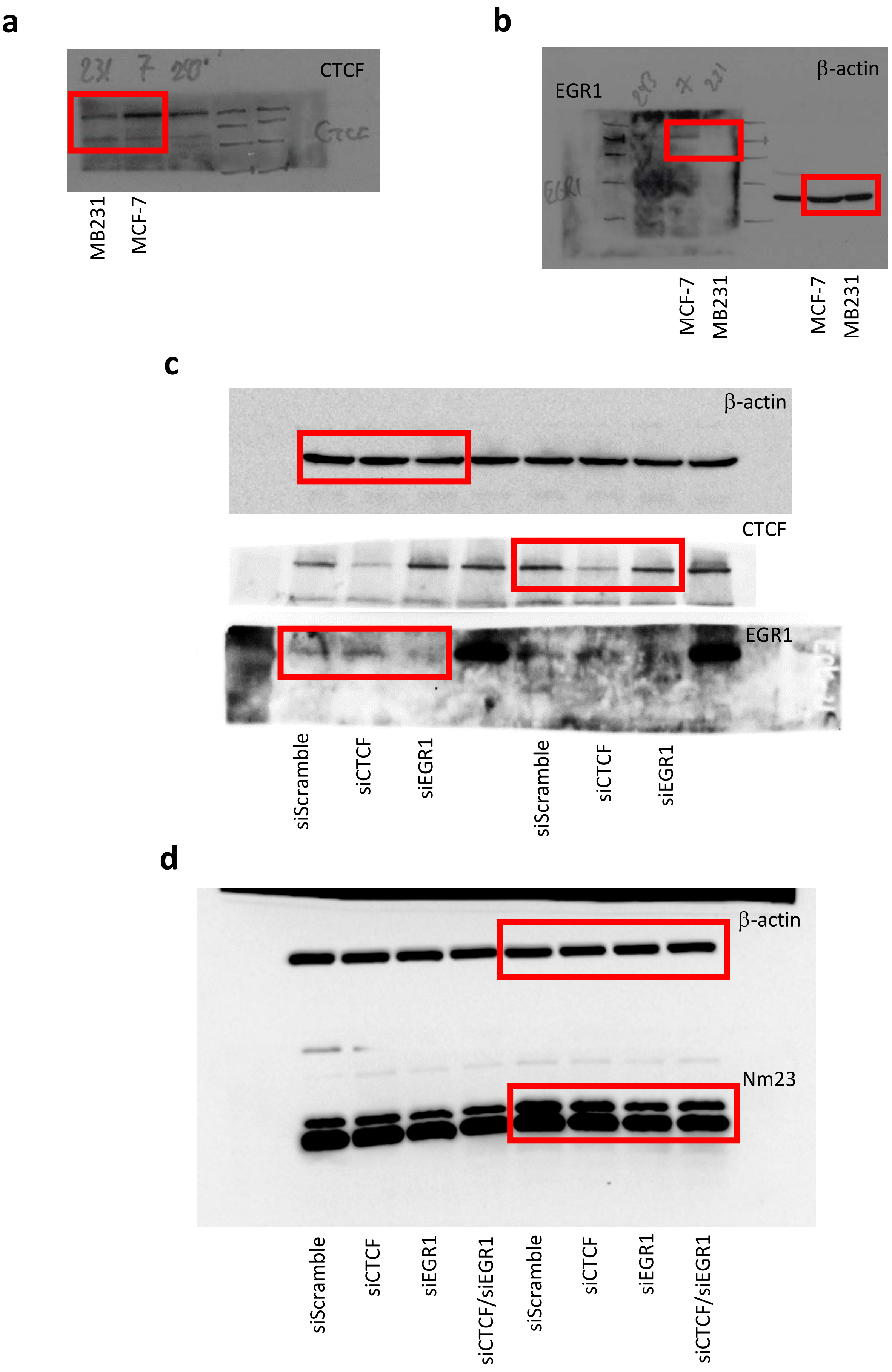

Supplementary Figure S7. Uncropped original version of Western blots (2). (**a,b**) Endogenous expression of CTCF and EGR1 in breast cancer cell lines, **Fig. 4a**. (**c**) siRNA-knockdown of CTCF and EGR1 in MCF-7 cells, **Fig. 7a**. (**d**) Nm23-H1 expression upon CTCF and EGR1 knockdown in MCF-7 cells, **Fig. 7d**.

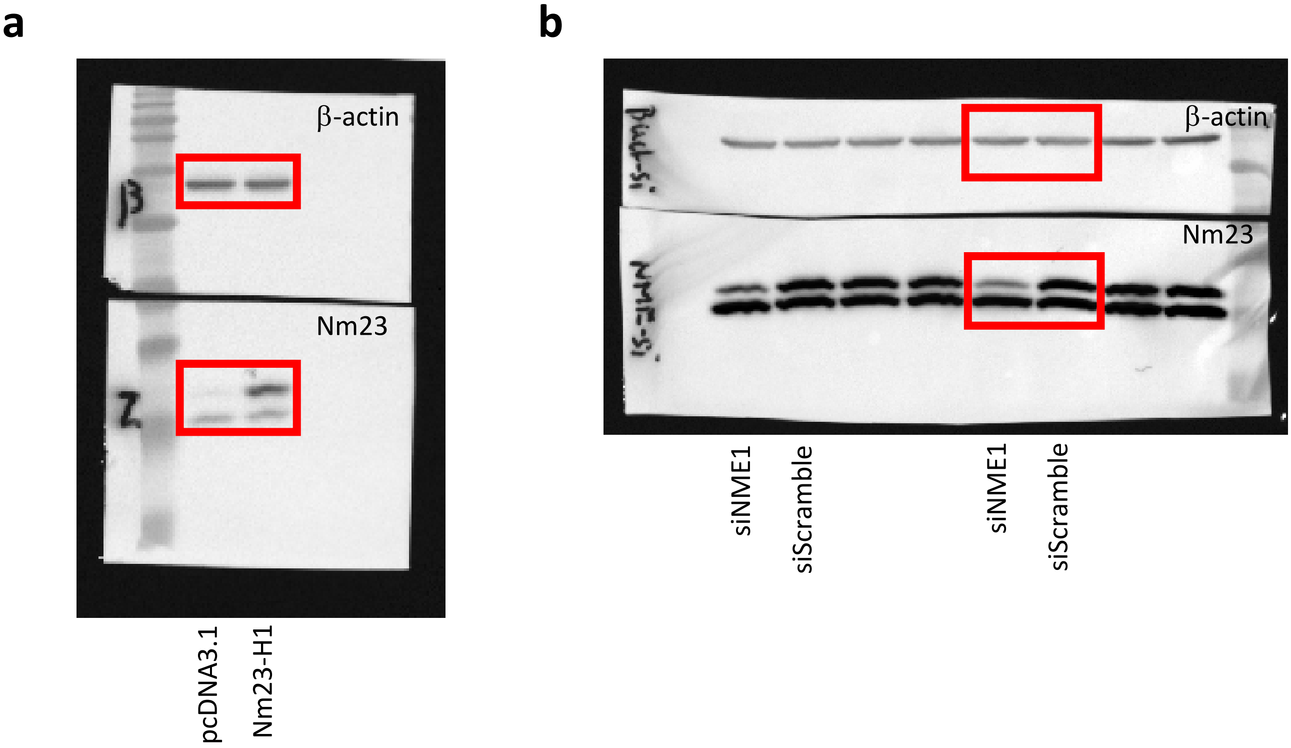

Supplementary Figure S8. Uncropped original version of Western blots (3). (**a**) Transient expression of Nm23-H1 in MDA-MB-231 cells, Supplementary Figure **S4**. (**b**) Knockdown of Nm23-H1 by siRNA in MCF-7 cells, Supplementary Figure **S5**.

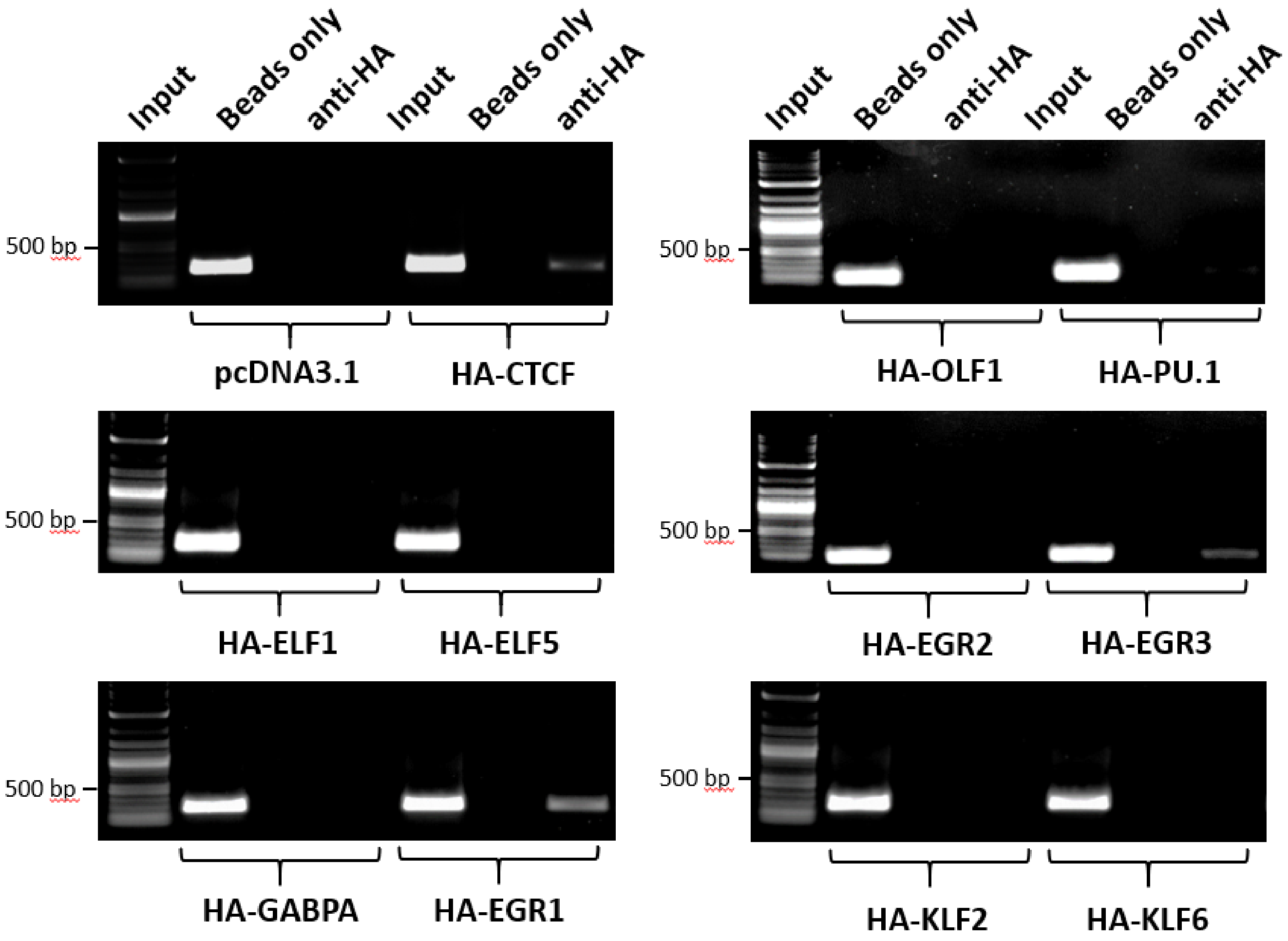

Supplementary Figure S9. Unprocessed original version of agarose gels. Transcription factors binding to Nm23-H1 promoter in MDA-MB-231 cells, **Fig. 3d**.

	Gene	Primer sequence
***NME1* promoter constructs**	(1291/1)	F: 5’-TTACTCGAGAGTCTCGCTCTGTCACCCAGGCT-3’<br>R: 5’-AGTAAGCTTGCACGGAACGCTTCTGCG-3’
	(930/1)	F: 5’-TTACTCGAGCTGGCCCTAGGTCCTGAGGATT-3’<br>R: 5’-AGTAAGCTTGCACGGAACGCTTCTGCG-3’
	(657/1)	F: 5’-CGGCTCGAGCTGTATTAGCATCTCACAACATAAGATTTGGC-3’<br>R: 5’-AGTAAGCTTGCACGGAACGCTTCTGCG-3’
	(372/1)	F: 5’-TGACTCGAGCTAGAATAATGCGTCCACGTAGTAGGT-3’<br>R: 5’-AGTAAGCTTGCACGGAACGCTTCTGCG-3’
	(244/1)	F: 5’-ATTCTCGAGGTGTGAGCGCCACCTCTC-3’<br>R: 5’-AGTAAGCTTGCACGGAACGCTTCTGCG-3’
	(109/1)	F: 5’-TGACTCGAGAGAAAGTTGTTCCCTAGAATGACTGCCTACT-3’<br>R: 5’-AGTAAGCTTGCACGGAACGCTTCTGCG-3’
	(61/1)	F: 5’-TTACTCGAGGCGAGCGGTGTTCTGCAAAATG-3’<br>R: 5’-AGTAAGCTTGCACGGAACGCTTCTGCG-3’
	(1291/930)	F: 5’-TTACTCGAGAGTCTCGCTCTGTCACCCAGGCT-3’<br>R: 5’-CGCAAGCTTGCAAATAGTTTTTAGACGACACCAAAAGACC-3’
	(1291/657)	F: 5’-TTACTCGAGAGTCTCGCTCTGTCACCCAGGCT-3’<br>R: 5’-CGCAAGCTTATTTTGCACAAGGCATGACATCATCAC-3’
	(1291/372)	F: 5’-TTACTCGAGAGTCTCGCTCTGTCACCCAGGCT-3’<br>R: 5’-CGCAAGCTTGCCCTAGGGATAAAACAGTATGCTC-3’
	(1291/244)	F: 5’-TTACTCGAGAGTCTCGCTCTGTCACCCAGGCT-3’<br>R: 5’-AGCAAGCTTGTTGCGTGAAGTCCAAAGAGTGC-3’
	(1291/109)	F: 5’-TTACTCGAGAGTCTCGCTCTGTCACCCAGGCT-3’<br>R: 5’-AGTAAGCTTGCAGTTTAACTTCCGGCGCTAG-3’
	(1291/61)	F: 5’-TTACTCGAGAGTCTCGCTCTGTCACCCAGGCT-3’<br>R: 5’-GCTAAGCTTCCACGCTTCCTCTTGGGAGT-3’
	(930/657)	F: 5’-TTACTCGAGCTGGCCCTAGGTCCTGAGGATT-3’<br>R: 5’-CGCAAGCTTATTTTGCACAAGGCATGACATCATCAC-3’
	(657/372)	F: 5’-CGGCTCGAGCTGTATTAGCATCTCACAACATAAGATTTGGC-3’<br>R: 5’-CGCAAGCTTGCCCTAGGGATAAAACAGTATGCTC-3’
	(372/244)	F: 5’-TGACTCGAGCTAGAATAATGCGTCCACGTAGTAGGT-3’<br>R: 5’-AGCAAGCTTGTTGCGTGAAGTCCAAAGAGTGC-3’
	(244/109)	F: 5’-ATTCTCGAGGTGTGAGCGCCACCTCTC-3’<br>R: 5’-AGTAAGCTTGCAGTTTAACTTCCGGCGCTAG-3’
	(109/61)	F: 5’-TGACTCGAGAGAAAGTTGTTCCCTAGAATGACTGCCTACT-3’<br>R: 5’-GCTAAGCTTCCACGCTTCCTCTTGGGAGT-3’
**Others**	ELF1 cDNA	F: 5’- ATGGCTGCTGTTGTCCAACAGAACGACCTA-3’<br>R: 5’-AAAAGAGTTGGGTTCCAGCAGTTCGTTTTGTTTCA-3’
	OLF1 cDNA	F: 5’-ATGTTTGGGATTCAGGAAAGCATCCAAC-3’<br>R: 5’-TCACATAGGAGGAACAATCATGCCAGATATC-3’

Supplementary Table S1. Primers for the cloning of *NME1* promoter regions and cDNAs. *NME1* promoter construct (1291/1) indicates that the region from -1,291 bp to -1 bp upstream of TSS of *NME1* is cloned into pGL3-Basic. Other cDNAs are cloned into pcDNA3.1. F, forward primer. R, reverse primer.

Gene	Primer sequence	Amplicon size
**NME1**	F: 5’-AAGGAGATCGGCTTGTGGTTT-3’<br>R: 5’-CTGAGCACAGCTCGTGTAATC-3’	60
**GAPDH**	F: 5’-AAGTTGTCATGGATGACCTTGGC-3’<br>R: 5’-GGCGTCTTCACCACCATGGAG-3’	206

Supplementary Table S2. Primers for amplification of cDNA in qPCR reactions. F, forward primer. R, reverse primer.
